# Neuroinflammation and Anxiety-Related Pathology: Apoptosis, Pyroptosis, and the Preclinical Potential of Paeoniflorin

**DOI:** 10.3390/ijms27219549

**Published:** 2026-10-26

**Authors:** Chen Li, Qiumei Dong, Yajing Hou

**Affiliations:** School of Traditional Chinese Medicine, Inner Mongolia Medical University, Hohhot 010110, China

**Keywords:** anxiety disorders, neuroinflammation, apoptosis, pyroptosis, paeoniflorin, signaling pathways

## Abstract

Anxiety disorders have a multifactorial pathophysiology involving neurotransmitter signaling, stress-responsive neuroendocrine systems, and distributed neural circuits. Neuroinflammation has emerged as an important component of this network, although its contribution varies across anxiety phenotypes and experimental contexts. Apoptosis and pyroptosis may contribute to cellular injury in selected preclinical models, but their causal roles in human anxiety disorders remain incompletely established. This review examines inflammatory signaling and cell-death pathways relevant to anxiety-related pathology and critically evaluates the evidence for paeoniflorin (PF), a monoterpene glycoside derived from Paeonia species. Particular attention is given to the tryptophan–kynurenine pathway, regional differences across amygdala, hippocampal, and prefrontal networks, and interactions among the hypothalamic–pituitary–adrenal axis, glucocorticoid signaling, and peripheral-to-central immune communication. PF-related changes in inflammatory, apoptotic, and pyroptotic markers are considered alongside neurochemical, neuroendocrine, and behavioral outcomes, with direct PF findings distinguished from evidence involving other interventions or disease models. Available studies support further investigation of PF as a preclinical candidate, rather than establishing clinically meaningful anxiolytic efficacy. Limitations in model validity, causal inference, dosing, bioavailability, and biomarker validation are discussed to guide mechanistic research and future clinical translation.

## 1. Introduction

Anxiety disorders are characterized by excessive fear, worry, and avoidance that interfere with daily functioning. Their clinical presentations include generalized anxiety disorder, social anxiety disorder, panic disorder, and specific phobias, and their development reflects interacting genetic, environmental, and neurobiological factors [1,2]. Cognitive-behavioral therapy and pharmacological treatments, including selective serotonin reuptake inhibitors and serotonin–norepinephrine reuptake inhibitors, provide established treatment options [1]. Nevertheless, delayed improvement, incomplete remission, and tolerability problems motivate investigation of additional therapeutic approaches. Such approaches should be evaluated within a multifactorial framework rather than assuming that anxiety disorders arise from a single neurotransmitter deficiency or inflammatory abnormality.

Neuroinflammation is increasingly investigated as one contributor to anxiety-related pathology. In experimental systems, altered microglial responses and inflammatory signaling can influence synaptic function and stress-related behavior. For example, prefrontal microglial Toll-like receptor 2/4 (TLR2/4) signaling contributes to repeated social defeat-induced social avoidance, although this behavioral endpoint should not be equated with an anxiety disorder [3]. Clinical findings are less uniform: inflammatory alterations have been reported in subsets of patients, with associations varying by phenotype, sex, and study population [4]. Peripheral cytokine concentrations do not directly identify their cellular source or establish corresponding inflammatory changes in the brain. Neuroinflammation should therefore be considered within an interacting pathophysiological network rather than described as an almost universal initiating event. The broader literature also describes neuroinflammatory mechanisms in Alzheimer disease and other neurological or psychiatric conditions [5,6]. Such overlap supports investigation of shared pathways but argues against treating inflammatory markers as anxiety-specific.

The relationship between stress, neurotransmission, and inflammation is potentially bidirectional. Stress-related neuronal activity and endogenous danger signaling may initiate neuroimmune responses even in the absence of an overt peripheral inflammatory insult. Acute stress-induced adenosine triphosphate (ATP) release and P2×7 receptor-dependent inflammasome activation provide an experimental example of this process [7]. Inflammatory signaling can subsequently alter tryptophan metabolism through indoleamine 2,3-dioxygenase 1 (IDO1), while stress-responsive regulation of tryptophan 2,3-dioxygenase 2 (TDO2) provides an additional metabolic link [8]. Changes in kynurenine metabolites may influence serotonergic availability, glutamatergic signaling, and redox balance. These interactions provide plausible routes toward synaptic dysfunction and cell-death susceptibility, but do not establish neurotransmitter deficiency as a uniform upstream cause of neuroinflammation in anxiety disorders.

Apoptosis and pyroptosis are distinct forms of regulated cell death that may connect inflammatory and oxidative stress to cellular injury. Their relevance to anxiety must be interpreted in relation to the affected cell populations and circuits, because inflammatory signaling can also modify neurotransmission and plasticity without producing overt cell loss. Paeoniflorin (PF), a monoterpene glycoside isolated from Paeonia species, has shown anti-inflammatory and cytoprotective effects in several experimental systems, including modulation of microglial pyroptosis-associated signaling and protection of neural progenitor cells against oxidative injury [9,10]. These findings motivate evaluation of PF in anxiety-related models, but evidence from depression, ischemia, peripheral tissues, or isolated cells cannot independently establish an anxiolytic mechanism or clinical efficacy.

This review integrates neuroinflammatory, neurochemical, neuroendocrine, and regional circuit mechanisms relevant to anxiety-related pathology, with apoptosis and pyroptosis as its central cellular focus. It then evaluates what PF studies actually demonstrate, distinguishes concurrent biochemical and behavioral changes from causal mediation, and separates anxiety-related findings from cross-model support. Finally, it considers the pharmacokinetic and methodological requirements for translating these preclinical observations into testable clinical hypotheses.

## 2. Molecular Mechanisms of Neuroinflammation in Anxiety Disorders

### 2.1. Microglial Responses and Pro-Inflammatory Signaling

Microglia maintain central nervous system (CNS) homeostasis and respond to changes in neuronal activity, tissue injury, and immune signals. Their responses may be adaptive or detrimental depending on the stimulus, timing, and local cellular environment. Increased concentrations of pro-inflammatory cytokines have been reported in subsets of patients with anxiety disorders, although findings vary across anxiety phenotypes, biological compartments, and study populations [4]. Blood or cerebrospinal fluid measurements alone cannot establish microglial activation or identify the brain region responsible for a behavioral phenotype.

Experimental stress can engage microglial pattern-recognition and purinergic signaling. TLR-dependent pathways influence nuclear factor-κB (NF-κB)-associated inflammatory transcription, whereas inflammasome activation can promote caspase-1-dependent processing of pro-IL-1β and pro-IL-18. Prefrontal microglial TLR2/4 signaling has been implicated in stress-induced social avoidance, and ATP–P2×7-related mechanisms have been linked to stress-responsive inflammasome activation and anxiety-like behavior in specific models [3,7,11]. These observations support model-dependent involvement of microglia without implying that the same pathway initiates all anxiety disorders. TLR4/NF-κB-related responses have also been investigated in ethanol-exposed adolescent hippocampus and inflammatory pain models [12,13]. Experiments involving plumbagin, fluoxetine, panaxytriol, and trefoil factor 3 demonstrate that cytokine production by stimulated microglia is pharmacologically modifiable [14,15,16,17]. These are cellular or cross-model observations, and the fluoxetine findings do not by themselves demonstrate that serotonin deficiency initiates inflammation. P2×7 inhibition has additionally been examined in obese rats subjected to an immune challenge, with inflammatory, neurogenic, and behavioral outcomes [18].

Microglial states cannot be adequately represented by a fixed pro-inflammatory M1 versus anti-inflammatory M2 dichotomy [19]. These labels may summarize selected experimental marker profiles, but inflammatory, phagocytic, metabolic, and reparative functions can coexist. Interpretation should therefore emphasize the measured cell populations, mediators, and functional outcomes. Likewise, increased NLR family pyrin domain-containing 3 (NLRP3) expression or cytokine release indicates inflammatory activity but does not by itself demonstrate pyroptotic death; gasdermin processing and membrane injury require separate assessment [20,21]. For example, an ATF5-dependent mitochondrial unfolded protein response limited cytokine secretion in activated BV2 cells [22]. Conditioned medium from TLR4-stimulated microglia reduced oligodendrocyte precursor proliferation without inducing cell death, while effects on mature oligodendrocytes differed [23]. These results illustrate why inflammatory activation cannot be equated with a single uniformly cytotoxic state.

Sustained inflammatory signaling may increase oxidative stress and alter neuronal or astrocytic function, while neuronal activity and endogenous danger signals can modify microglial responsiveness. The resulting interactions are best understood within region-specific networks rather than as a uniform inflammatory process throughout the brain. Neuron–microglia experimental systems exposed to lanthanum and studies involving Gualou Guizhi Granule have linked inflammatory microglial responses to neuronal apoptosis-related outcomes [24,25]. Chronic central IL-1β administration and IL-10 treatment have also been studied alongside glial, neurotrophin, apoptosis-associated, and behavioral measures [26]. These observations provide cross-model support for inflammation–cellular injury interactions rather than evidence that all anxiety-related cytokine changes cause neuronal death.

### 2.2. Regional Inflammatory Dysfunction in Interconnected Anxiety-Related Circuits

The behavioral effects of neuroinflammation depend on where and in which cells it occurs. The amygdala, hippocampus, and prefrontal cortex participate in complementary aspects of threat evaluation, contextual processing, and behavioral regulation. Cytokine measurements in these regions become more informative when linked to synaptic, cellular, and circuit-level outcomes.

Within the amygdala, the basolateral amygdala (BLA) integrates sensory and contextual information relevant to threat. In a lipopolysaccharide (LPS) model, local inflammatory changes were accompanied by enhanced presynaptic glutamate release, altered excitatory/inhibitory balance, and increased neuronal excitability, together with anxiety- and depressive-like behavior [27]. Such findings support a route from inflammation to heightened threat-related circuit activity. However, increased glutamatergic excitation should not automatically be described as excitotoxic neuronal death: demonstrating excitotoxicity requires evidence of injury in addition to elevated excitability. Persistent excitation and inflammatory signaling may reinforce each other, but a complete self-sustaining loop must be established experimentally. Chronic-stress experiments involving fatty acid amide hydrolase manipulation further connect endocannabinoid signaling, BLA microstructure, and anxiety-like behavior [28]. This is complementary evidence for stress-sensitive amygdala plasticity, not a direct test of an inflammatory or PF mechanism.

The hippocampus contributes to contextual discrimination, memory, and stress regulation. Inflammatory signaling can affect hippocampal plasticity and adult neurogenesis; IL-1β-dependent suppression of neurogenesis has been demonstrated in a stress model [29]. Separately, experimental manipulation of adult hippocampal neurogenesis has linked it to stress buffering and hypothalamic–pituitary–adrenal (HPA)-axis regulation [30,31]. Together, these studies provide a mechanistic basis for investigating how inflammation may impair contextual threat processing and increase anxiety generalization. They do not establish that reduced neurogenesis explains every anxiety phenotype. More directly, microglial NKAα1–P2×7-related signaling has been linked to ventral hippocampal neuronal and neurogenic alterations in a stress-induced anxiety model [11]. Peripheral LPS challenge has been studied using time-resolved hippocampal inflammatory and gene-expression measures alongside sickness and anxiety-like outcomes [32]. Hippocampal KDM6B/VGLL4 experiments provide an additional example of regionally investigated inflammatory signaling in an LPS anxiety-like model [33]. By contrast, a CRS study linked impaired hippocampal insulin signaling to memory deficits, illustrating that stress-responsive hippocampal changes need not produce an anxiety-specific endpoint [34].

Prefrontal networks regulate threat-related behavior through projection- and subregion-dependent interactions with the amygdala and other structures. Their contribution cannot be reduced to a universal inhibitory action on the amygdala. Circuit manipulation has demonstrated that particular prefrontal–amygdala projections regulate anxiety and fear, with effects depending on their targets [35]. Stress-related prefrontal microglial signaling can alter neuronal function and behavior, providing inflammatory evidence that complements these circuit studies [3]. However, the combined literature should not be presented as a single experimentally verified cytokine-to-prefrontal-to-amygdala causal chain. In an allergic-asthma model, inhaled corticosteroid pretreatment partly attenuated inflammatory, behavioral, and oscillatory abnormalities in the medial prefrontal cortex (mPFC)–amygdala network [36]. Exercise-related cortical FMRP/mTOR responses and manipulation of mPFC Dnmt3a have independently been investigated in stress/anxiety-like paradigms [37,38]. These studies reinforce regional functional interpretation, while their distinct interventions cannot be used as evidence of PF selectivity.

These regions operate as interconnected compartments. Amygdala threat reactivity may interact with impaired hippocampal contextual processing and altered prefrontal regulation, while hippocampal and hypothalamic stress feedback adds a neuroendocrine dimension. The behavioral consequences depend on the affected cell populations, projection-defined circuits, temporal profile, and disease context, rather than cytokine identity alone. Regional inflammatory changes may bias network function toward anxiety-related phenotypes without being uniquely specific to anxiety; depressive and cognitive symptoms may arise from shared pathways. This framework also distinguishes a PF effect measured in a particular region from evidence that PF selectively targets that region.

**Figure 1:**
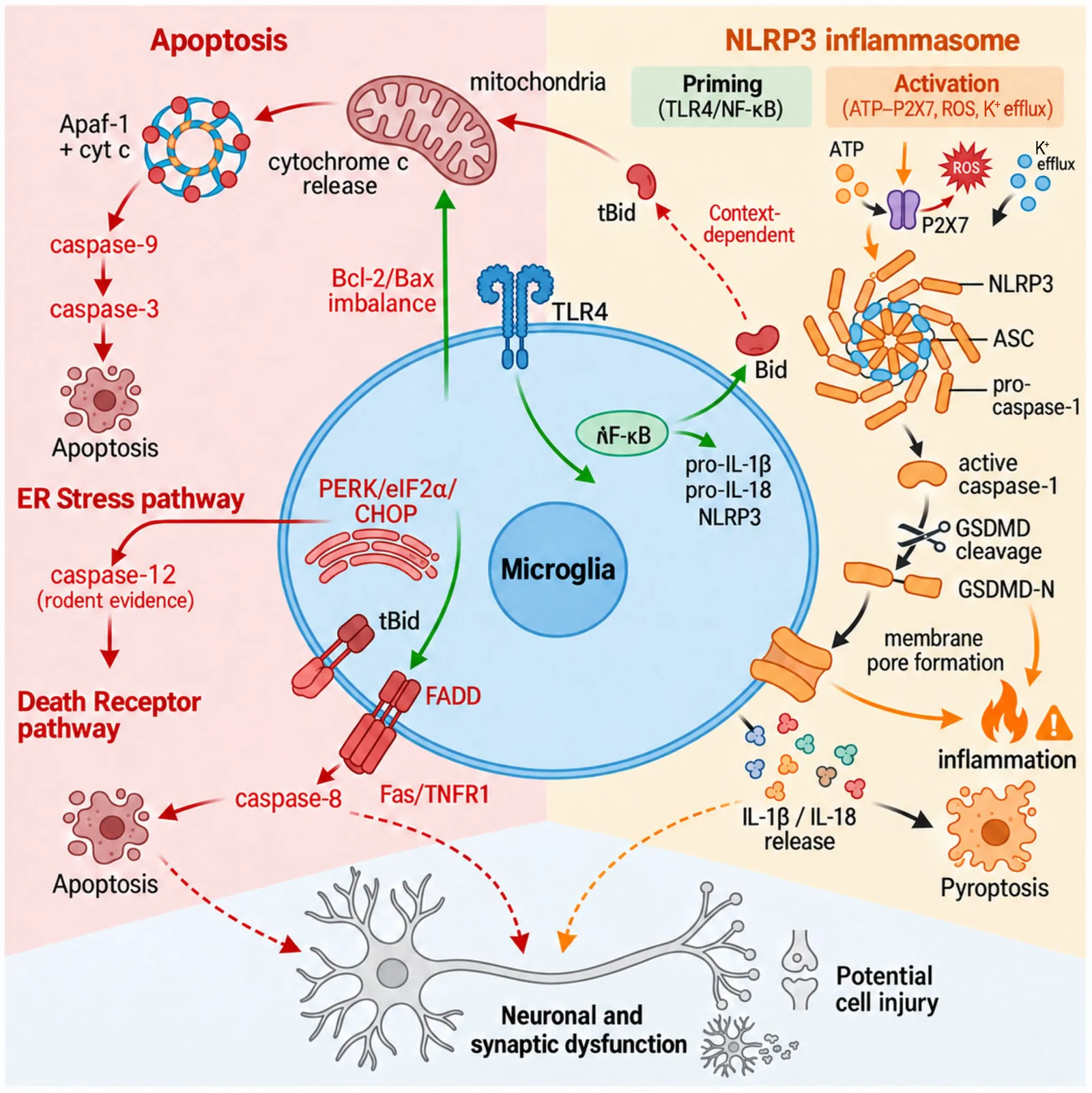
Cellular pathways potentially linking neuroinflammation to injury in anxiety-related experimental models.

Stress-related danger signals and inflammatory stimuli can engage microglial Toll-like receptor/NF-κB and ATP–P2×7 signaling in selected experimental models [3,7,11]. The diagram summarizes mitochondrial, endoplasmic reticulum (ER), and death receptor-associated routes toward apoptosis, together with NLRP3/caspase-1/GSDMD signaling that can produce pyroptotic membrane injury [20,21,39,40,41] (Figure 1). Reactive oxygen species (ROS), ATP, and potassium efflux may contribute to inflammasome activation. Caspase-8/BID connects death receptor signaling to mitochondria, whereas caspase-1/BID-dependent apoptosis has been demonstrated under conditions including GSDMD deficiency [42,43]. These are context-dependent cellular mechanisms; their occurrence, cellular localization, and behavioral contribution must be established in each anxiety-related model. Caspase-12 evidence is primarily rodent-derived and should not be generalized to human anxiety disorders [44,45].

### 2.3. Oxidative Stress as an Interface Between Inflammation and Cellular Injury

Oxidative stress provides a potential interface between inflammatory signaling, altered neurotransmission, and regulated cell death [46]. Excess reactive oxygen species can perturb mitochondrial function, calcium handling, and protein homeostasis, whereas damaged cellular components may amplify danger signaling. These interactions can increase susceptibility to mitochondrial apoptosis or inflammasome-associated injury under sufficiently severe or sustained conditions. They should not be interpreted as evidence that all inflammatory changes in anxiety produce neuronal death. Kynurenine metabolites provide a specific example of this interface: 3-hydroxykynurenine has induced neuronal death with apoptotic features in culture, and quinolinic acid has produced oxidative and excitotoxic injury in experimental systems [47,48]. In astrocytic systems, bradykinin- and IL-1β-associated signaling engaged NADPH oxidase 2-derived ROS and MMP-9-related migration [49,50]. A hippocampal neuronal culture study linked NOX2/ROS to NLRP1-associated aging responses; NLRP1 should not be conflated with NLRP3 [51]. ROS sources also depend on cell population: a multiple-sclerosis model implicated monocyte-derived macrophage mitochondrial ROS rather than a uniform microglial NOX2 mechanism [52]. Experimental interventions involving L-F001, artemisinin, and tetrahydrocurcumin have examined redox/inflammatory responses in different glial or disease settings [53,54,55]. A high-fat-diet/chamomile study assessed anxiety-like behavior alongside cerebral oxidative and inflammatory measures, whereas Netrin-3 research addressed diabetic cognitive impairment [56,57]. A pellitorine study investigated CRS-associated cognitive and depression-like outcomes together with ferroptosis, showing that stress-related cellular injury is not restricted to apoptosis and pyroptosis [58]. The following sections place these cellular mechanisms within their upstream metabolic and neuroendocrine context.

### 2.4. Tryptophan–Kynurenine Metabolism at the Neurotransmitter–Immune Interface

The tryptophan–kynurenine pathway provides a potential metabolic link between stress, immune activity, and neurotransmission. Its engagement does not require infection or an externally administered inflammatory stimulus. Psychological stress can increase extracellular ATP and activate P2×7-dependent inflammasome signaling [7]. Stress-responsive neuroendocrine signals may also influence tryptophan metabolism, although the responsible enzymes and cellular responses vary across tissues and brain regions [8].

IDO1 catalyzes the initial oxidation of tryptophan toward kynurenine formation. Inflammatory regulation includes well-established IFN-γ-driven IDO1 induction, with TNF-α enhancing this response in particular cellular contexts [59]. Other cytokines should not be assumed to exert equivalent direct effects on IDO1. TDO2 also contributes to tryptophan catabolism and offers a stress-sensitive route that is not exclusively dependent on IDO1. Acute stress studies reveal glial and tissue-specific enzyme regulation, arguing against a uniform microglial response [8].

Changes in this pathway may affect serotonergic substrate availability and downstream glutamatergic or redox signaling. Nevertheless, enhanced tryptophan catabolism does not automatically demonstrate reduced synaptic serotonin transmission. Different neural and glial populations have distinct metabolic capacities, and their downstream metabolite profiles depend on the physiological and inflammatory context [8,60]. Among relevant products, 3-hydroxykynurenine is associated principally with oxidative injury, whereas quinolinic acid can promote N-methyl-D-aspartate (NMDA) receptor-mediated excitotoxicity [47,48]. Kynurenic acid can antagonize the NMDA receptor-associated glycine site [61]. Oxidative and excitotoxic stress may thereby increase susceptibility to apoptosis or inflammasome-associated injury, without proving kynurenine-driven pyroptosis in anxiety.

Chronic restraint stress (CRS)-related changes in gut and brain kynurenine metabolism, together with intervention using an IDO inhibitor, support involvement in selected stress-associated behavioral phenotypes [62]. Clinical evidence remains heterogeneous: a social anxiety disorder study reported increased kynurenic acid-related measures without generalized elevations in tryptophan catabolism or pro-inflammatory cytokines [63]. Peripheral metabolite changes do not directly establish CNS metabolism or activation of a neurotoxic branch. The pathway is therefore considered a potential mechanistic interface rather than a universal causal mechanism across anxiety disorders.

### 2.5. HPA-Axis Dysregulation, Glucocorticoid Signaling, and Peripheral-to-Central Neuroimmune Communication

The hypothalamic–pituitary–adrenal (HPA) axis connects stress appraisal to endocrine and immune regulation. Hypothalamic corticotropin-releasing hormone (CRH) promotes pituitary adrenocorticotropic hormone (ACTH) release and subsequent adrenal glucocorticoid secretion. Cortisol is the principal glucocorticoid assessed in humans, whereas corticosterone is commonly measured in rodent studies. Glucocorticoid receptor (GR)-dependent feedback limits HPA activity, with hippocampal and hypothalamic networks contributing to its regulation [31,64]. Adult hippocampal neurogenesis has also been implicated in experimental regulation of stress feedback [31].

Glucocorticoids normally exert anti-inflammatory actions, so HPA activation should not be portrayed as inevitably increasing cytokine production. The outcome depends on exposure duration, tissue, and timing relative to an immune challenge. Experimental glucocorticoid exposure can prime subsequent microglial inflammatory responses, while chronic stress-associated peripheral glucocorticoid resistance has been linked to impaired inflammatory regulation [65,66]. These mechanisms provide routes through which stress and inflammatory signaling can reinforce one another, although their direction and magnitude vary between models.

Peripheral immune signals can influence the brain through neurovascular and endothelial communication without requiring prior blood–brain barrier (BBB) breakdown. In repeated social defeat, interactions between IL-1β-producing monocytes and IL-1R1-expressing brain endothelial cells have been implicated in neuroimmune responses and anxiety-like behavior [67]. Under selected conditions, changes in endothelial tight-junction integrity and permeability may further amplify peripheral-to-central communication. Stress-related vascular changes have been demonstrated in a depression-related nucleus accumbens model, whereas early life social isolation was associated with amygdala Claudin-5 loss, BBB abnormalities, and anxiety-like behavior specifically in female mice [68,69]. These findings establish regional and sex-dependent examples rather than BBB damage as a prerequisite for anxiety. Lung–brain inflammatory communication and altered vascular permeability have been examined in septic-survivor mice [70]. In aged mice with dextran sulfate sodium (DSS)-induced colitis, fucoidan treatment was evaluated alongside barrier, neuroinflammatory, and anxiety/depressive-like outcomes [71]. These infection and colitis paradigms illustrate peripheral-to-central interactions under specific conditions, rather than establishing BBB leakage as a universal anxiety trigger.

Inflammatory signaling may also modify GR function rather than physically block cortisol receptors. Cell experiments have demonstrated IL-1α/p38-associated impairment of GR signaling [72]. Human evidence links chronic stress-associated peripheral glucocorticoid resistance to altered inflammatory regulation, but this does not directly demonstrate impaired central HPA feedback [66]. Peripheral immune glucocorticoid resistance, regional GR expression, and endocrine feedback are related yet distinct measurements. Where central feedback or immune glucocorticoid sensitivity is impaired, reduced restraint of stress and inflammatory responses could favor their persistence. This proposed contribution to chronification requires direct evaluation rather than inference from receptor expression alone.

HPA phenotypes are heterogeneous across anxiety-related conditions; sex and symptomatic state influence cortisol stress reactivity [73]. Sustained hypercortisolemia should therefore not be assumed in every disorder. Moreover, GR-dependent dopaminergic adaptations demonstrated in social aversion do not necessarily explain anxiety, because one circuit study found anxiety and fear-memory measures unaffected [74]. Overall, these observations support an interacting neuroendocrine–immune framework, although the complete feedback loop has not been demonstrated within a single model or across all anxiety disorders. This framework informs the interpretation of PF findings without presupposing uniform HPA normalization.

## 3. Apoptotic Pathways Relevant to Anxiety-Related Cellular Injury

### 3.1. Mitochondrial Apoptosis

The mitochondrial apoptotic pathway integrates cellular stress through BCL-2-family proteins. When pro-apoptotic signaling overcomes anti-apoptotic restraint, BAX/BAK-dependent mitochondrial outer membrane permeabilization permits cytochrome c release [75]. Cytosolic cytochrome c supports APAF1 apoptosome formation and caspase-9 activation, followed by executioner caspase activation [39]. Changes in mitochondrial membrane potential may accompany injury but should not be treated as the universal initiating event or as a specific demonstration of apoptosis.

Inflammatory, oxidative, and excitotoxic stress can converge on mitochondrial homeostasis. Kynurenine-associated oxidative or NMDA-related injury provides a plausible upstream contribution, as discussed in Section 2.4 [47,48]. Nevertheless, this established cellular pathway should be distinguished from its disease-specific relevance. The Bcl-2/Bax ratio, mitochondrial potential, and caspase-3 abundance are informative endpoints, but none alone establishes the extent, cell type, or behavioral consequences of neuronal apoptosis in anxiety. Stronger inference requires complementary structural and functional measures, ideally together with pathway-specific intervention.

### 3.2. Endoplasmic Reticulum Stress and Apoptotic Susceptibility

The endoplasmic reticulum (ER) supports protein folding and calcium homeostasis. Its unfolded protein response is initially adaptive; sustained or unresolved stress can shift signaling toward injury. PERK/eIF2α/ATF4-related induction of CHOP provides one route by which prolonged ER stress interacts with apoptotic susceptibility. Experimental CHOP overexpression has been linked to reduced Bcl-2 expression, glutathione depletion, and enhanced oxidant injury [40]. ER and mitochondrial stress should thus be viewed as interacting processes rather than obligatorily independent pathways.

Species differences are especially relevant to caspase-12. ER-associated caspase-12-dependent injury was demonstrated in mouse experimental systems, whereas human CASP12 polymorphism produces different protein forms with distinct biological implications [44,45]. Rodent caspase-12 findings therefore cannot be presented as a universal human anxiety mechanism. Likewise, GRP78 or CHOP changes indicate stress-response engagement but require additional evidence to establish apoptotic execution. Evidence from toxic injury, peripheral tissues, or other neurological conditions supplies mechanistic support, not direct proof of ER stress-mediated neuronal death in anxiety patients. Doxorubicin-exposed rats treated with dl-3-n-butylphthalide were assessed for hippocampal ER/oxidative stress and anxiety/depression-like behavior [76]. In a single prolonged stress model, gypenoside XVII was investigated in relation to prefrontal microglial PERK/CHOP signaling, apoptosis, and fear-extinction outcomes [77]. These studies make ER stress relevant to selected injury or stress paradigms while providing no direct evidence of a PF-mediated ER mechanism in primary anxiety.

### 3.3. Death Receptor-Mediated Extrinsic Apoptosis

Death receptors can couple extracellular signals to caspase-8 activation, but receptor-specific signaling architecture matters. Fas signaling involves a death-inducing signaling complex, whereas TNFR1 can generate sequential signaling complexes with inflammatory, survival, or death outcomes depending on cellular conditions [41]. TNF-α exposure therefore does not inevitably produce neuronal apoptosis, and TNFR1 signaling should not be described as identical to Fas-associated complex formation.

Activated caspase-8 can engage downstream apoptotic caspases and cleave BID to generate truncated BID, providing a connection to mitochondrial cytochrome c release [42]. This is an established molecular bridge between extrinsic and intrinsic apoptosis. Its activation in anxiety-related circuits, however, must be demonstrated rather than inferred from cytokine elevation. Studies in kidney, endometrial, or cancer cells cannot establish microglial TNF-α-driven death receptor apoptosis in the amygdala. The pathway remains relevant as a candidate mechanism of inflammation-associated cellular injury, with region- and cell-specific validation needed to connect it to anxiety-related behavior. The molecular dependence of extrinsic apoptosis can vary across cellular systems: endometrial stromal-cell work implicated ERK/NF-κB-associated survival, while lung- and colon-cancer experiments investigated VDAC1/caspase-8 and BAX dependence [78,79,80]. These non-neural studies support interpretation of pathway architecture, not regional neuronal death in anxiety.

## 4. Inflammasome Signaling, Pyroptosis, and Their Relationship to Anxiety-Related Pathology

### 4.1. NLRP3 Inflammasome Assembly and Activation

NLRP3 inflammasome signaling is commonly considered in terms of priming and activation. Priming increases the availability of inflammatory components, including NLRP3 and pro-IL-1β, whereas activation promotes assembly with ASC and pro-caspase-1 [81,82]. Danger signals associated with ATP–P2×7 signaling, potassium efflux, and cellular stress can participate in this process. Activated caspase-1 processes pro-IL-1β and pro-IL-18 and can cleave GSDMD, linking cytokine maturation to a potential membrane pore-forming response [20,21]. Post-translational control provides an additional regulatory level: pharmacological experiments targeting BRCC3-associated NLRP3 deubiquitination suppressed inflammasome activity in inflammatory disease models [83]. This establishes a regulatory mechanism distinct from merely reducing NLRP3 abundance.

Experimental evidence supports inflammasome involvement in selected anxiety-related contexts. Early life LPS exposure increased forebrain inflammasome-associated proteins alongside adolescent anxiety-like behavior, and stress-responsive purinergic signaling has been linked to inflammasome activity and anxiety-related hippocampal changes [7,11,84]. These studies differ in initiating stimulus, brain region, and behavioral phenotype. Findings from diabetic encephalopathy or depression models should be identified as cross-model support rather than relabeled as direct anxiety evidence [85,86]. Restraint-stress studies involving agmatine measured hippocampal/PFC inflammasome and cytokine responses, while sodium aescinate research examined dorsal raphe NLRP3-related changes in post-traumatic stress disorder (PTSD)-like rats [87,88]. NU9056 studies evaluated LPS-associated encephalopathy and behavioral outcomes; Bifico studies addressed estrogen-deficiency-associated depression/anxiety-like phenotypes [89,90]. Separately, intracerebral hemorrhage experiments implicated mitochondrial ROS in NLRP3 activation and neurological injury [91]. These findings broaden the experimental context without establishing one shared causal sequence or PF-specific dependence. Tabersonine has also been investigated in depression-related paradigms for suppression of NLRP3-associated responses [92]. This is evidence involving a different compound and behavioral domain and does not establish PF-dependent anxiolysis.

Inflammasome activation and pyroptosis are not interchangeable. Increased NLRP3, ASC, cytokines, or caspase-1-related signals may occur without demonstrating gasdermin-mediated lytic death. Inference about pyroptosis requires convergent evidence for gasdermin processing, membrane injury, and the identity of the affected cells. Moreover, an intervention that changes both inflammasome markers and behavior supports association unless dependence is tested directly. Inflammasome-associated inflammatory signaling may affect synapses and neuronal activity without requiring extensive cell loss.

### 4.2. GSDMD-Mediated Membrane Injury

Inflammatory caspase cleavage of GSDMD releases its pore-forming N-terminal domain. Foundational studies established GSDMD cleavage and the membrane pore-forming activity of this domain [20,21]. In an appropriate cellular context, this process promotes membrane permeabilization and inflammatory mediator release and can culminate in pyroptotic death. Gasdermin expression alone, however, is not sufficient evidence of pore formation or completed lysis. Gasdermin-family structural synthesis further distinguishes pore-forming domains and inflammatory-caspase cleavage from protein abundance alone [93]. Primary studies have demonstrated additional regulation through GSDMD palmitoylation and SYVN1-associated ubiquitination [94,95]. These post-translational mechanisms require their own assays and cannot be inferred from reduced GSDMD expression.

The relevance of this mechanism to anxiety depends on the model and cell population. An arsenic exposure study examined neuronal pyroptosis-associated signaling together with anxiety-related phenotypes, whereas traumatic brain injury research has investigated astrocytic pyroptosis in the amygdala [96,97]. These are exposure- or injury-associated settings, not proof of a universal mechanism in primary anxiety disorders. Bioinformatic and oligodendrocyte experiments relating GSDMD to Alzheimer-associated pathology provide additional cross-disease evidence, rather than direct evidence in anxiety [98]. Astrocyte-specific NLRP3 deletion has been examined in mild-TBI depression-like outcomes and hemorrhagic-shock/resuscitation anxiety-like paradigms [99,100]. Other experimental work investigated microglial pyroptosis-associated responses in neuropathic pain and oxygen–glucose-deprivation injury of brain vascular pericytes [101,102]. These studies emphasize that the relevant cell population may be astrocytic, microglial, or neurovascular, depending on the insult.

Cell death can also have context-dependent effects. A study of specific microglial subsets found that lytic death was required to prevent atypical behavior in mice, arguing against a uniformly harmful interpretation of all microglial death [103]. Translational discussion should therefore specify the gasdermin, cell type, anatomical location, and experimental conditions, and separate inflammatory responses from demonstrated tissue injury.

### 4.3. Context-Dependent Crosstalk Between Apoptosis and Pyroptosis

Apoptotic and pyroptotic machinery can interact without constituting a fixed sequential cascade. In the absence of GSDMD, caspase-1 signaling has been shown to induce BID-dependent apoptotic responses [43]. Conversely, apoptotic caspase-mediated cleavage can inactivate GSDMD and alter the balance between cell-death programs [104]. These findings demonstrate flexibility in cell-fate regulation, rather than proving that pyroptosis always precedes mitochondrial apoptosis in anxiety-related neurons.

Inflammatory mediator release and cellular damage may also provide indirect links: cytokines can influence surrounding cells, while damage-associated signals may amplify immune activation. The contribution of these interactions to persistent circuit dysfunction requires model-specific testing. Parallel reductions in caspase-1 and caspase-3, if observed, would not alone establish causal coupling, synergy, or interruption of a complete apoptosis–pyroptosis loop. A post-stroke emotional-disorder study assessed dl-3-n-butylphthalide together with PANoptosis-associated markers, but coordinated changes in such markers do not establish a complete causal cell-death sequence [105]. A separate primary study demonstrated extracellular-vesicle transfer of GSDMD pores to bystander cells [106]. The corresponding commentary discusses this as a mechanism for propagating inflammatory cell death [107]. Its relevance to anxiety circuits and PF-mediated protection remains untested. Table 1 summarizes the relevant mechanisms and distinguishes established cellular processes from their incompletely defined relevance to anxiety disorders.

## 5. Anti-Inflammatory and Neuroprotective Effects of Paeoniflorin

### 5.1. Chemical Identity and Pharmacokinetic Considerations

PF is a monoterpene glycoside with the molecular formula C23H28O11. Pure PF must be distinguished from total glucosides of paeony, multi-component formulas, structural derivatives, and unrelated compounds with similar abbreviations. These preparations may differ substantially in pharmacology and systemic exposure [108]. Previous reviews summarize PF pharmacology across neurological, cardiovascular, and renal disease models [109]. Such breadth motivates cross-model comparison but does not establish an anxiety indication.

Low oral bioavailability is a major obstacle to translation. A rat pharmacokinetic study reported absolute oral bioavailability of approximately 3–4% under its tested conditions [110]. Intestinal transport and microbiota-associated metabolism may contribute to exposure variability; an experimental study also examined benzoic acid as a PF-related metabolite [111]. These findings do not establish which parent compound or metabolite drives effects in particular anxiety-related brain regions.

Formulation studies provide potential routes to improve exposure. A PF–phospholipid complex increased oral exposure in rats, whereas MDCK-MDR1 monolayer experiments used as an in vitro BBB model examined PF transport and P-glycoprotein-related interactions [112,113]. The latter study also tested transport modulation by co-administered constituents. Neither improved systemic exposure nor transport in an in vitro barrier model independently establishes adequate unbound brain concentrations or anxiolytic efficacy in vivo. Translation requires pharmacokinetic measurements aligned with the dose, route, model, and pharmacodynamic outcomes, as discussed in Section 9.2. Glycyrrhiza protein-based PF nanoparticles have also been investigated for oral exposure and analgesic performance [114]. This provides formulation support, while neither analgesia nor enhanced absorption constitutes evidence of anxiolytic efficacy or regional CNS delivery.

### 5.2. PF-Associated Modulation of Microglial Inflammatory Responses

Direct PF studies support modulation of microglial inflammatory responses in selected experimental systems. In a reserpine-associated depression study, PF was investigated in mice and N9 microglial cells in relation to CASP-11/GSDMD-associated pyroptotic signaling [9]. A subsequent study using chronic unpredictable mild stress (CUMS)-exposed mice and LPS/ATP-stimulated BV2 cells linked PF effects to SIRT1, NF-κB, and NLRP3-associated responses, with an inhibitor experiment supporting SIRT1 involvement [115]. These findings provide mechanistic support in depression-related settings rather than direct validation in human anxiety. Additional PF experiments in LPS-exposed microglia and organotypic hippocampal slices examined cytokine production and inflammation-associated neuronal injury [116,117]. These studies provide direct PF cellular evidence, although their endpoints do not establish a complete anxiety mechanism.

PF has also been associated with reduced hippocampal inflammatory signaling in an LPS-induced depression-like model [118]. Interpretation should prioritize actual measurements, such as cytokines, pathway proteins, cell morphology, and behavioral endpoints, instead of describing a universal M1-to-M2 conversion. Microglial marker changes do not, on their own, establish functional reprogramming or cell-specific mediation of behavioral improvement. Amyloid-stimulated microglial studies examined PF-related inflammatory and chemotactic responses, while an amnesic mouse study investigated inflammatory, antioxidant, and cognitive outcomes [119,120]. An EAE study reported PF interaction with α-enolase (ENO1) and modulation of microglial/macrophage glycolytic and inflammatory responses [121]. The latter supplies target-oriented support in autoimmune CNS disease, not proof of ENO1-mediated anxiolysis.

Combination-treatment evidence requires separate attribution. A BV2 study tested PF, ferulic acid, and atractylenolide III alone and in combination [122]. Its PF-alone arm informs single-compound effects, whereas findings specific to the combined treatment cannot establish that every observed effect is produced by PF alone. Studies of harmine, fustin, hydrogen sulfide, quercetin, puerarin, and magnoflorine provide comparative neuroimmune evidence, but their effects must not be attributed to PF [123,124,125,126,127,128]. The PF evidence discussed in this review is restricted to studies that actually administered PF.

### 5.3. Antioxidant and Mitochondrial Cytoprotection

PF-related antioxidant and mitochondrial effects have been reported across neural and non-neural experimental systems. In hydrogen peroxide-exposed neural progenitor cells, PF preserved viability and modified apoptosis-associated proteins, with PI3K/Akt inhibition reducing its protective effect [10]. Nuclear factor erythroid 2-related factor 2 (Nrf2)-associated responses have also been investigated in Schwann cells, and an LPS-induced depression-like study assessed PF-related Nrf2/heme oxygenase-1 (HO-1) signaling and mitochondrial responses [118,129]. These studies support cytoprotective actions under their respective conditions. Peripheral PF studies provide complementary evidence in cigarette smoke-associated lung injury, oxidatively challenged melanocytes, and LPS-induced liver injury [130,131,132]. The melanocyte study implicated JNK/Nrf2/HO-1-associated responses, illustrating that the direction of MAPK modulation depends on the experimental context. These results cannot establish central antioxidant target engagement from systemic dosing alone.

Such findings should not be generalized to selective preservation of hippocampal mitochondria in anxiety or to direct regulation of specific respiratory-chain complexes without corresponding experiments. Reduced ROS and improved membrane potential may attenuate upstream cellular stress but do not by themselves establish prevention of mitochondrial outer membrane permeabilization or interruption of inflammasome-dependent death. The functional relevance of these changes requires alignment with cell identity, brain exposure, and circuit or behavioral outcomes.

## 6. PF-Associated Modulation of Apoptotic Signaling: Evidence and Relevance to Anxiety

### 6.1. Mitochondrial Apoptosis and Survival Signaling

The anti-apoptotic potential of PF is supported by studies that actually administer the compound. In oxidatively challenged neural progenitor cells, PF improved viability and altered Bcl-2/Bax- and caspase-associated measures; pharmacological inhibition implicated PI3K/Akt in cellular protection [10]. PF has also been investigated in neonatal hypoxic–ischemic brain injury [133]. These studies offer neural cytoprotection evidence, but neither constitutes a CRS anxiety experiment nor establishes hippocampal neuronal survival as a mediator of anxiolysis. PF-related suppression of oxidative and apoptosis-associated injury has also been examined in neomycin-exposed hair cells and cisplatin-exposed spiral ganglion neurons [134,135]. H9C2 reperfusion experiments investigated SIRT1/PINK1/parkin-associated mitochondrial autophagy [136]. Schwann-cell proteomics investigated PF-related mitochondrial thioredoxin responses, but the accompanying in vivo neuropathy experiment administered total glucosides of paeony capsules rather than purified PF [137]. These preparation and tissue differences limit direct extrapolation to anxiety circuits.

Compound identity is critical when evaluating the anti-apoptotic literature. Findings involving PF derivatives or multi-component preparations should not be assigned to the parent compound without a separate PF treatment arm. The present synthesis therefore distinguishes pure PF findings from related preparations and considers the model-specific limits of each experimental result.

Current findings support investigation of PF effects on mitochondrial stress and apoptotic susceptibility. They do not establish that PF stabilizes Bcl-2 directly, prevents cytochrome c release in anxiety-related hippocampal neurons, or acts through the same pathway across stress models. Establishing such mechanisms requires region- and cell-specific measurements together with tests of dependence.

**Figure 2:**
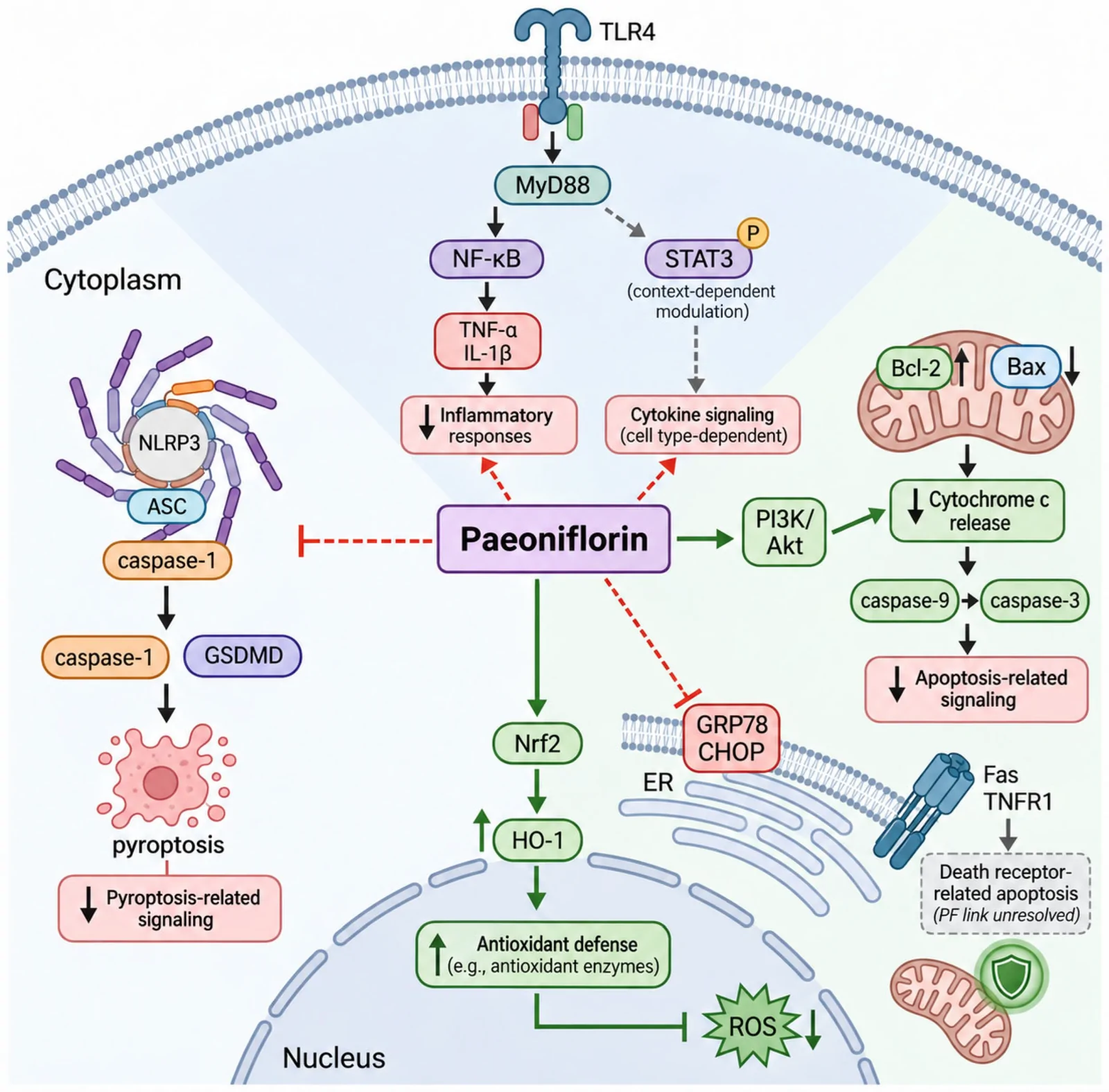
Experimentally reported PF-associated signaling responses and their proposed relevance to cellular protection.

PF-associated inflammatory and cytoprotective responses have been reported in different experimental systems. Microglial/depression-related studies examined CASP-11/GSDMD- and SIRT1/NF-κB/NLRP3-associated signaling, while neural progenitor experiments implicated PI3K/Akt in protection against oxidative injury [9,10,115]. ER/mitochondrial and antioxidant findings include hepatic, retinal, endothelial, and Schwann-cell models [129,138,139,140]. These observations support context-dependent pathway involvement rather than simultaneous validation of all depicted mechanisms in anxiety-related neurons (Figure 2). Direct PF binding to the NLRP3 ATP-binding domain, inhibition of BID-mediated crosstalk in anxiety circuits, and uniform STAT3 suppression are not established by these studies. The proposed connections require model-specific target engagement and functional validation.

### 6.2. ER Stress-Associated Cytoprotection

PF has been studied in cellular systems involving ER stress or related injury. In D-galactosamine/TNF-α-challenged L02 hepatic cells, PF was associated with protection against apoptosis and changes in ER stress-related measures [138]. Additional studies examined protection in retinal pigment epithelial cells exposed to all-trans-retinal and inflammatory/ER signaling in endothelial cells [139,140]. These experiments differ in cell type, insult, and pathway readouts, and should not be merged into a single established PERK/eIF2α/CHOP mechanism in anxiety-related neurons.

The evidence supports cross-model investigation of PF effects on protein and calcium homeostasis. It does not yet demonstrate that ER stress suppression mediates anxiety-like behavioral improvement. Confirmation would require brain exposure measurements, identification of the responsive neural cells, complementary apoptosis endpoints, and perturbation of the proposed ER signaling pathway.

### 6.3. Death Receptor-Related Signaling: Current Evidence Limits

Inflammatory cytokines can influence receptor-mediated survival and death signaling, but reducing TNF-α is not equivalent to demonstrating inhibition of Fas or TNFR1 apoptotic complexes. Although PF-related protection has been investigated in TNF-α-associated hepatic injury, this does not establish reduced death-inducing signaling complex formation, caspase-8 activity, or BID cleavage in the amygdala [138].

Direct evidence that PF suppresses death receptor-mediated apoptosis within anxiety-related circuits remains limited. Accordingly, extrinsic apoptosis is retained as a potentially relevant cellular pathway, while PF-specific involvement is treated as an unresolved question. Regional receptor measurements and pathway perturbation would be necessary before attributing PF-associated behavioral improvement to this mechanism.

## 7. PF-Associated Modulation of Inflammasome and Pyroptosis Signaling

### 7.1. NLRP3-Associated Inflammatory Signaling

PF has been associated with reduced inflammasome-related responses in depression-related experimental systems. In CUMS-exposed mice and LPS/ATP-stimulated BV2 cells, PF-related changes in SIRT1, NF-κB, NLRP3, caspase-1, and GSDMD-related measures were examined, and SIRT1 inhibition attenuated selected protective effects [115]. These findings support pathway involvement under the tested conditions. The caspase-11/GSDMD findings from a separate reserpine-associated study are discussed separately below rather than assigned to the same canonical NLRP3/caspase-1 mechanism [9]. Reduced NF-κB-associated transcription may influence inflammasome priming, while improved redox or mitochondrial homeostasis may affect activation-related stress. However, changes in pathway proteins do not independently establish a direct PF–NLRP3 interaction. Molecular docking is hypothesis-generating and should be distinguished from biochemical binding assays, target engagement, and mutational validation. The available studies do not justify the specific claim that PF binds the NLRP3 ATP-binding domain to prevent oligomerization in the PFC of anxiety models.

Additional PF studies provide cross-model support for links among cellular stress and inflammasome-associated responses. In CUMS mice and corticosterone-challenged HT22 cells, PF-related mitochondrial autophagy and NLRP3 responses were examined, with autophagy inhibition attenuating protective effects [141]. PF studies have also reported spinal NLRP3-related effects in neuropathic pain and submandibular gland Nrf2/HO-1–NLRP3 responses in experimental Sjögren’s syndrome [142,143]. These findings should be identified by their model, tissue, cell type, and endpoints; they do not establish a uniform anti-pyroptotic mechanism for anxiety disorders. Other PF studies examined NLRP3-related regulation in pulmonary arterial hypertension, diabetic liver injury, fatty liver disease, and diabetic cardiomyopathy [144,145,146,147]. Their proposed upstream mechanisms include SIRT1 regulation, TXNIP-associated responses, STING-related signaling, and AMPK/Nrf2 pathways, respectively. An alcohol-exposure mouse study also reported PF-associated depressive-like, inflammatory, and monoamine-related changes [148]. These results provide tissue- and model-dependent PF support without validating the same pathway in anxiety.

### 7.2. GSDMD-Associated Responses and Direct-Target Evidence

The reserpine-associated study in mice and N9 microglia examined PF in relation to caspase-11/GSDMD-dependent pyroptotic signaling, while a CUMS/BV2 study investigated SIRT1–NF-κB–NLRP3/caspase-1-associated responses [9,115]. These are distinct experimental mechanisms. Their findings support modulation of pyroptosis-associated responses, but do not demonstrate that PF physically blocks the caspase-1–GSDMD interaction. Neither docking predictions nor parallel changes in cleaved proteins substitute for an experimentally verified interaction mechanism.

Direct-target evidence has been reported in a different biological setting. An ANIT-induced cholestatic liver injury study used target screening, cellular thermal shift analysis, and surface plasmon resonance to support PF binding to GSDMD, alongside cellular and animal protection [149]. This finding is relevant to PF pharmacology but derives from hepatic models rather than microglia or anxiety-related circuits. It should therefore be classified as peripheral mechanistic evidence, with central target engagement and behavioral mediation still requiring investigation.

The interpretation of PF as a potential regulator of GSDMD-associated injury is thus stronger than a wholly untested hypothesis, but narrower than an established CNS anxiolytic mechanism. Confirmation in relevant neural cells would require exposure-matched binding and functional assays, complemented by selective manipulation of GSDMD.

### 7.3. Potential Convergence of Apoptotic and Pyroptotic Responses

PF-related effects on oxidative stress, inflammatory signaling, and cellular survival may converge on more than one injury pathway. Nevertheless, the studies discussed here use different models, tissues, and endpoints. They do not collectively demonstrate that PF simultaneously blocks a causal apoptosis–pyroptosis cascade within anxiety-related circuits.

Published microglial and depression-related findings and neural progenitor cell survival experiments support PF-associated modulation of cellular stress in different experimental settings [9,10,115]. Parallel changes in apoptotic and inflammasome-associated markers suggest potential convergence, but do not establish synergy or interruption of a single causal cascade. Demonstrating these mechanisms would require time-resolved measurements, evidence that the pathways interact in the same identified cells or circuit, and selective blockade or rescue of the relevant signaling steps.

PF is therefore discussed as a candidate modulator of convergent cellular stress and injury processes. Whether this convergence contributes to anxiety-related behavioral improvement remains a mechanistic question, not a conclusion established by reductions in caspase abundance alone. Table 2 summarizes the direct PF findings and their interpretive limits.

**Figure 3:**
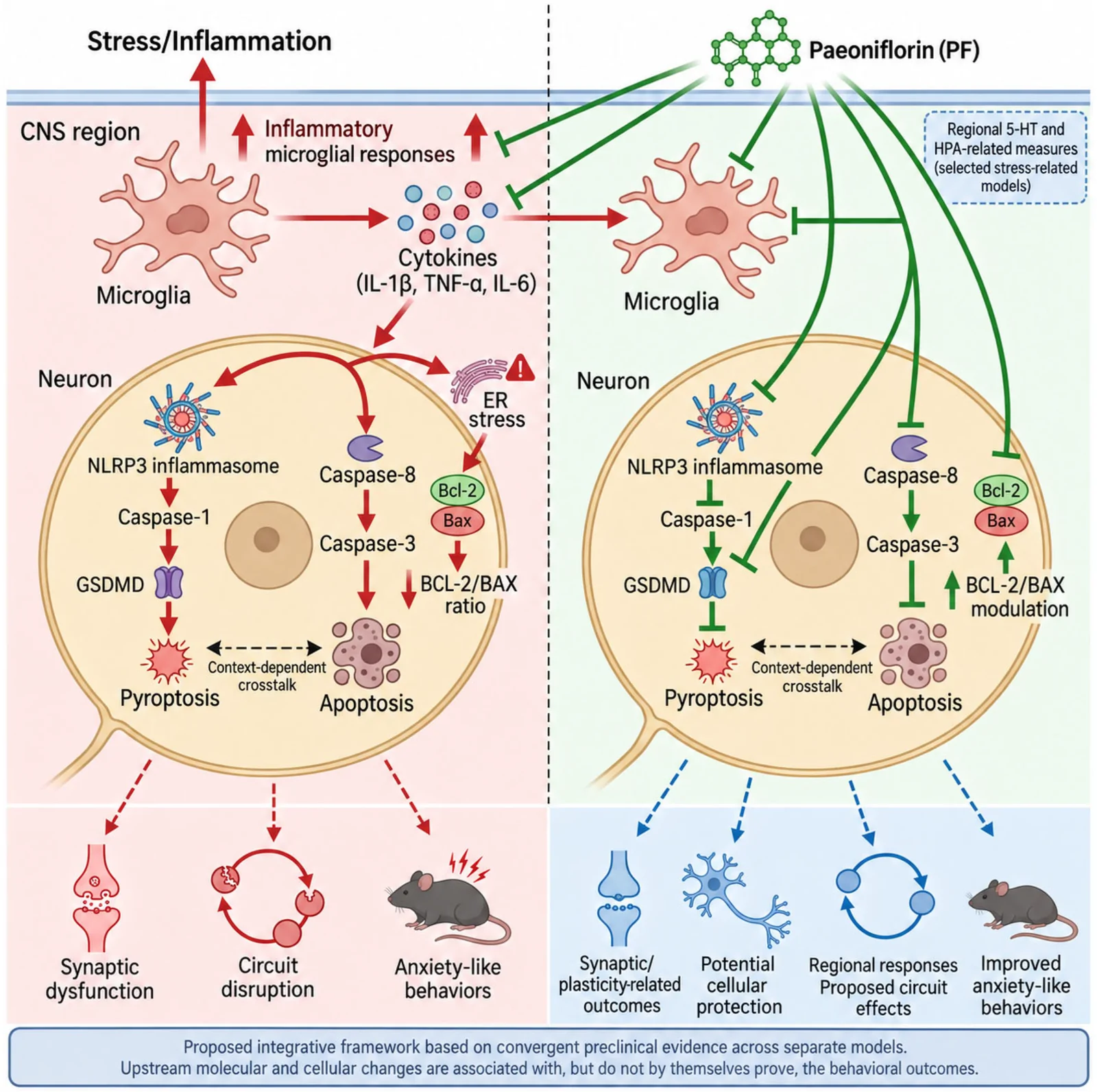
Proposed integration of PF-associated neuroimmune, cellular, and functional responses.

PF findings across separate experimental systems suggest potential connections among inflammatory signaling, oxidative stress, cellular protection, and behavioral responses [9,10,115,118]. The diagram represents an integrative framework, not a complete mechanism demonstrated within one anxiety model (Figure 3). Molecular protection may influence surviving cells and network function as well as susceptibility to injury. Neurotransmitter, HPA-axis, and behavioral measurements are therefore needed to evaluate the functional relevance of these changes. Regional observations do not establish PF selectivity for the hippocampus, PFC, or amygdala; reduced inflammatory or cell-death markers do not alone establish anxiolytic mediation or clinical efficacy.

## 8. Direct PF Findings in Anxiety-Related and Supportive Experimental Models

### 8.1. Stress-Related Models: Behavioral, Serotonergic, and HPA Outcomes

Stress-based and immune-challenge models differ in their initiating mechanisms. CRS and single prolonged stress (SPS) paradigms begin with stress-related neural and endocrine responses, whereas systemic LPS initiates innate immune activation and subsequent CNS/endocrine responses. These models can engage interacting pathways but should not be treated as interchangeable representations of anxiety disorders. SPS is commonly used for PTSD-related phenotypes; its anxiety-like outcomes provide supportive evidence without equating PTSD with all primary anxiety disorders. Comparative stress-model studies have investigated spinosin, Jie-Yu-He-Huan capsule, and γ-oryzanol using behavioral and neurotrophic or monoaminergic measures [156,157,158]. Bithionol, oxotremorine, and Moringa seed lectin studies likewise examined inflammatory or neurochemical correlates of behavioral responses in stress paradigms [159,160,161]. These interventions help define testable functional endpoints but cannot establish the efficacy or mechanism of PF.

In SPS-exposed rats, Qiu et al. reported PF-associated improvement in elevated-plus-maze and contextual-fear outcomes, together with regional serotonin (5-HT)/5-hydroxyindoleacetic acid (5-HIAA) and circulating HPA-related changes [150]. This constitutes direct PF neurochemical evidence in a stress-related model. Regional tissue concentrations do not establish synaptic serotonergic transmission, and their concurrent improvement with behavior does not prove mediation.

Chen et al. also investigated PF in SPS-exposed rats, including dexamethasone suppression and regional corticosteroid receptor measurements [151]. The study identified time- and region-dependent endocrine/receptor patterns, including enhanced feedback at an early model stage [151]. Its findings are therefore described as modulation of HPA feedback and regional GR/MR expression, rather than uniform correction of HPA hyperactivity or restoration of globally impaired feedback. Together, these studies link PF treatment to neurochemical and neuroendocrine outcomes, while preserving the distinction between concurrent association and causal mechanism.

### 8.2. Regional Inflammatory Findings: PVN Anxiety Evidence and Hippocampal Cross-Model Support

In sleep-deprived male mice, PF reduced anxiety-like behavior and inflammatory measures in the hypothalamic paraventricular nucleus (PVN), with additional neuronal and pathway analyses supporting regional involvement [152]. This provides direct PF evidence in an anxiety-related paradigm localized to the PVN. It does not independently establish corresponding effects in hippocampal, PFC, or BLA circuits, nor does PVN localization alone establish correction of the entire HPA feedback system.

An LPS-induced depression-like mouse study reported PF-associated changes in hippocampal inflammation, neuronal and synaptic measures, and antioxidant signaling [118]. CUMS-related hippocampal and microglial inflammasome responses have also been examined [115]. These experiments provide regional cross-model support for PF-related neuroimmune modulation. Their depressive-like endpoints should remain explicitly distinguished from anxiety-like behavior. PF studies in chronic constriction injury and nerve-cuff-associated pain/depression models also examined spinal or hippocampal inflammatory responses [162,163]. These regional observations concern pain and depressive-like endpoints and should remain separate from direct PF anxiety evidence.

General regional circuit studies, including BLA electrophysiological findings involving harmine, help explain why inflammatory changes might affect threat processing [123]. They are not direct PF findings. Consequently, current PF evidence is distributed across several regions and models, including the PVN, while direct comparative evidence across the hippocampus, PFC, and BLA remains limited. Regional effects should not be interpreted as pharmacological regional selectivity without comparative exposure and response measurements.

### 8.3. Mechanistic Interpretation and Priorities for Validation

The available PF evidence includes concurrent neurochemical and behavioral changes, pharmacological tests of pathway involvement in selected models, and direct-target work outside the CNS. These are different levels of evidence. In particular, genetic NLRP3 or GSDMD experiments involving other interventions cannot establish PF-specific dependence. Regional neuronal analyses in a PF experiment likewise do not establish genetic dependence of its cell-death or behavioral effects.

Future studies should test whether identified inflammatory or cell-death processes are required for PF-related behavioral effects. Selective pathway inhibition, conditional genetic manipulation, and rescue experiments could distinguish mediation from parallel responses. Electrophysiology, regional neurotransmitter sampling, and endocrine feedback testing should be matched to brain exposure and behavioral measures. Tests must also distinguish anxiety-like improvement from changes in locomotion, sedation, sickness, pain, or memory. Table 2 reports what has actually been measured; Section 10.4 integrates its possible functional meaning.

## 9. Preclinical Limitations and Prospects for Translation

### 9.1. Model Validity and Causal Inference

PF has preclinical potential, but its evidence base spans heterogeneous models with different initiating insults and behavioral domains. SPS, sleep deprivation, CUMS, systemic LPS, neural cell cultures, and peripheral injury models each address only part of anxiety-related biology. PTSD-related or depressive-like results should therefore be treated as supportive evidence, while primary anxiety relevance requires further testing. No study discussed here establishes clinically meaningful anxiolytic efficacy of pure PF in patients.

Behavioral improvement must be interpreted alongside locomotor activity and other potential confounders. Open-field and elevated-plus-maze results can be affected by sickness, sedation, altered exploration, or motor performance. Similarly, fear-conditioning outcomes may reflect memory changes as well as threat processing. Molecular outcomes require equivalent care: tissue 5-HT concentration is not synaptic transmission, GR expression is not feedback function, and increased inflammasome components are not proof of pyroptotic death. Studies that observe concurrent changes cannot establish that one mediates another. Priorities include blinded and randomized experiments, both sexes, clinically relevant comparators, exposure–response assessment, and replication across independent models. Pathway-specific manipulation should determine whether neuroimmune and cell-death changes are necessary for PF-related behavior rather than merely associated with it.

### 9.2. Dose, Route, Bioavailability, and Exposure–Response Relationships

PF regimens differ markedly across studies. Examples include 10–20 mg/kg intraperitoneally in SPS rats, 40 mg/kg orally in another SPS experiment, and 15–60 mg/kg intraperitoneally in sleep-deprived male mice [150,151,152]. An LPS-induced depression-like study used 20–80 mg/kg/day by oral gavage [118]. These values describe experimental regimens, not an established therapeutic range for anxiety. Route, treatment timing, formulation, species, and active metabolites must be considered before comparing efficacy or extrapolating to humans.

Rat studies report low absolute oral bioavailability, approximately 3–4% in one investigation, while another study examined intestinal microbial metabolism and PF-related benzoic acid formation [110,111]. Brain pharmacology therefore cannot be inferred from an administered dose alone. Future work should quantify plasma and regional brain exposure, distinguish parent PF from metabolites, and compare achievable unbound concentrations with those active in cellular assays. Improvement in systemic exposure after formulation changes does not necessarily establish CNS delivery [112]. Human-equivalent dose calculations may inform initial planning but cannot determine clinical dosing without pharmacokinetic and safety studies.

### 9.3. Safety and Clinical Development

Experience with total glucosides of paeony or PF-containing formulas should be separated from evidence for purified PF. Human pharmacokinetic work on intravenous XueBiJing demonstrates that PF exposure can be characterized within a multi-component preparation, but does not establish the safety, bioavailability, or efficacy of orally administered pure PF for anxiety [164]. The accompanying constituents, route, indication, and exposure profile preclude direct equivalence.

Translation requires characterization of the intended product, purity, formulation, metabolites, repeated-dose tolerability, and interaction potential. Initial studies should establish exposure and safety before evaluating behavioral efficacy. Subsequent controlled trials would need predefined anxiety phenotypes, validated symptom measures, functional outcomes, and adequate assessment of tolerability and durability. A proposed anti-inflammatory mechanism should guide measurable hypotheses; it cannot substitute for a clinically meaningful benefit. Available preclinical findings support further evaluation rather than an assumption that improved bioavailability alone will permit clinical application.

### 9.4. Candidate Biomarkers and Response Stratification

Candidate biomarker domains include peripheral inflammatory measures, tryptophan–kynurenine metabolites, HPA-related endocrine measures, and PF/metabolite exposure. CRP and cytokine panels may help characterize systemic inflammatory status, while tryptophan, kynurenine, and downstream metabolite ratios could assess metabolic responses. However, inflammatory associations vary across anxiety populations, and peripheral kynurenine findings do not necessarily reflect CNS pathway activity [4,63]. Because IDO1 and TDO2 both contribute to tryptophan catabolism, a peripheral kynurenine/tryptophan ratio should be interpreted as an indirect metabolic measure rather than an isolated assay of IDO1 activity [8,153].

HPA assessment could include time-standardized glucocorticoid sampling and, where justified, dynamic feedback measures, with attention to sex, sampling time, medication, and symptomatic state. Cortisol reactivity is heterogeneous across anxiety-related conditions; a single concentration cannot establish glucocorticoid resistance [73]. Likewise, TSPO-related imaging requires careful interpretation because its relationship to inflammatory activation differs across species and cellular contexts [165]. A longitudinal generalized anxiety disorder (GAD) psychotherapy study measured diffusion-based spectral imaging restricted-fraction changes alongside symptom improvement [166]. These magnetic resonance imaging (MRI) measures are indirect and do not establish a cellular inflammatory mechanism or a PF response biomarker. In a rat colitis study, ex vivo [11C]PBR28 biodistribution changes were not equivalently detected by positron emission tomography (PET) imaging, illustrating measurement and sensitivity limits [167].

These measures represent research candidates, not validated predictors of PF response. Pharmacokinetic biomarkers establish exposure, pharmacodynamic biomarkers assess treatment-related biological change, and predictive biomarkers must demonstrate differential clinical benefit associated with a pretreatment characteristic. Future trials should test these roles explicitly and determine whether biological changes track symptom and functional improvement.

## 10. Integrating PF-Associated Signaling with Functional Outcomes

### 10.1. NF-κB and Context-Dependent STAT3 Responses

NF-κB-associated transcription offers a plausible point of convergence for inflammatory responses examined in PF studies. Depression-related experiments have linked PF to reduced microglial or hippocampal inflammatory measures [9,115,118]. These results should be interpreted according to the cells and stimuli studied rather than used to infer a single direct molecular target. PF-related NF-κB/inflammatory responses have additionally been examined in amyloid-stimulated C6 glial cells and LPS-associated myocardial injury [168,169]. The latter provides a peripheral comparison and does not establish a direct PI3K/Akt/ERK target in anxiety-related brain regions.

STAT3-related findings further illustrate context dependence. PF-associated STAT3 suppression has been investigated in glioma cells, whereas JAK2/STAT3 activation has been implicated in protection of oxygen–glucose-deprived PC12 cells [170,171]. These responses are not contradictory if cellular goals and stimuli differ, but they do not support universal inhibition of an NF-κB/STAT3 axis in anxiety-related neurons. The functional consequence of signaling modulation must be established in the relevant cell population and circuit.

### 10.2. Context-Dependent PI3K/Akt Signaling

In neural progenitor cells exposed to oxidative injury, PI3K/Akt inhibition reduced PF-associated protection, supporting pathway involvement in cellular survival [10]. Conversely, the sleep-deprivation PVN study reported PF-related modulation of abnormally elevated EGFR/PI3K/Akt-related signaling [152]. Thus, pathway activation and suppression may each be associated with benefit under different experimental conditions. Podocyte/diabetic kidney experiments reported PF interaction with VEGFR2 and reduced VEGFR2–PI3K/Akt-associated activity alongside autophagy and apoptosis outcomes [172]. This is target-oriented peripheral evidence that further argues against universal activation of PI3K/Akt by PF.

PI3K/Akt should be discussed as a context-dependent signaling domain, not a uniformly activated PF survival mechanism. Studies involving other plant extracts or unrelated compounds cannot establish PF-specific effects. Even within a PF study, altered phosphorylation is not evidence that the pathway mediates an anxiety-related behavioral response unless dependence is tested.

### 10.3. MAPK Signaling and Cellular Stress Responses

MAPK pathways participate in inflammatory, oxidative, and survival responses. PF studies in ischemic brain injury, retinal pigment epithelial injury, and toxicant-exposed hypothalamic neurons support modulation under their respective conditions [173,174,175]. Their different experimental settings preclude a universal rule that PF inhibits p38/JNK while necessarily activating ERK. Additional PF work examined hippocampal p38/JNK-associated responses after neonatal hyperoxia and spinal MAPK responses in chronic constriction injury [176,177]. In the latter study, PF and albiflorin were assessed separately, and their results should not be merged. Pancreatic β-cell and vascular smooth-muscle experiments provide further examples of PF-associated p38/JNK or ERK regulation outside the CNS [178,179]. Together, these studies support context-dependent modulation rather than a single direction of MAPK action across tissues.

These pathways interact with transcriptional and redox responses discussed in Section 5, Section 6 and Section 7. However, their roles in cell viability should be distinguished from circuit-level function and behavior. Functional integration requires testing how the measured molecular changes relate to neurotransmitter signaling, neuroendocrine regulation, and the affected anxiety-related networks, rather than assuming that fewer injury markers necessarily predict anxiolysis.

### 10.4. Neurotransmitter and Neuroendocrine Interfaces: From Molecular Protection to Behavioral Hypotheses

Inflammatory and cell-death pathways may influence behavior through changes in surviving cells, synaptic function, and stress-responsive networks. Therefore, anti-inflammatory or cytoprotective findings should be linked to functional endpoints rather than assumed to produce anxiolysis. Available PF studies offer several points of contact with neurotransmitter and neuroendocrine biology, but the strength and anatomical relevance of the evidence differ. Previous reviews discuss monoaminergic, neuroendocrine, and neuroprotective mechanisms in PF-related depression research and PF-containing formulas [180,181]. Those syntheses provide background, while the functional interpretation below prioritizes identifiable purified-PF experiments and their relevance to anxiety.

For serotonergic function, SPS research reported PF-associated changes in PFC and hippocampal 5-HT/5-HIAA alongside behavioral improvement [150]. These are regional neurochemical associations rather than proof of restored synaptic transmission or serotonergic mediation. Importantly, a study using TDO-overexpressing cells and stress-induced depression-like mice identified PF-related TDO inhibition and changes in hepatic tryptophan metabolism [153]. Thus, a PF–tryptophan metabolism connection has experimental support beyond a purely hypothetical link. Its extension to brain IDO1 activity, downstream neurotoxic metabolites, and anxiety-related circuits nevertheless requires direct investigation.

Glutamatergic evidence is more limited anatomically and behaviorally. In febrile-seizure and hippocampal culture experiments, PF influenced glutamate-related calcium responses, while the tested conditions did not demonstrate altered GABA production or release [154]. This does not establish normalization of amygdala excitatory/inhibitory balance. Reducing inflammatory or oxidative stress might preserve glutamate handling and synaptic function, but this remains a hypothesis requiring measurements of glutamate/GABA dynamics and identified BLA neuronal populations. The available evidence does not support a generalized GABAergic action of PF.

Dopaminergic claims require similar restraint. Intracerebroventricular PF administration altered regional monoamine metabolite measures in experimental rodents, but this invasive administration and non-anxiety setting do not establish normalization of mesolimbic dopamine function [155]. Neither these observations nor the HPA studies demonstrate a PF-specific sequence of restored feedback followed by dopaminergic normalization and anxiolysis. Such a sequence remains a candidate explanation and would require linked endocrine, dopamine-release, circuit, and behavioral measurements.

PF-associated modulation of HPA feedback and region-specific corticosteroid receptors in SPS rats provides a neuroendocrine connection, with effects interpreted according to model stage and receptor location [151]. As outlined in Section 2.5, interactions among stress, glucocorticoid sensitivity, and inflammation could connect these endocrine responses to cellular stress. However, the entire proposed endocrine–immune–neurotransmitter loop has not been demonstrated as a PF-mediated mechanism.

The functional evidence therefore ranges from concurrent association to mechanistic support in particular experimental systems. Causal mediation requires showing that disruption of the proposed mechanism abolishes or substantially alters the PF-related behavioral effect, ideally with rescue and control for nonspecific changes. The current findings provide testable explanations for preclinical behavioral improvement, but do not yet establish clinically meaningful anxiolytic efficacy in patients.

## 11. Conclusions

Neuroinflammation is one component of the interacting biological processes associated with anxiety-related pathology. Its contribution depends on phenotype, anatomical location, cell population, and experimental context. Stress-responsive danger signaling, tryptophan–kynurenine metabolism, glucocorticoid regulation, and peripheral-to-central communication provide plausible connections between inflammation and neurotransmitter or circuit dysfunction. These connections should not be treated as a universal, fully demonstrated causal loop. Apoptosis and pyroptosis may contribute to injury in selected models, while inflammatory effects on synapses and cellular function can occur without overt neuronal death.

Direct PF studies support modulation of inflammatory and cellular stress responses, with additional neurochemical, neuroendocrine, and behavioral findings in stress-related or sleep-deprivation paradigms. Evidence from depression, peripheral injury, and isolated cells provides complementary support but cannot establish regional selectivity, a common anxiolytic mechanism, or clinical efficacy. Effects involving other compounds, derivatives, or multi-component preparations must remain separately attributed.

PF should therefore be regarded as a candidate with preclinical therapeutic potential that warrants further evaluation. Priorities include exposure-matched mechanistic studies, region- and cell-specific validation, robust behavioral controls, and tests of causal mediation. Low oral bioavailability, uncertain contributions of metabolites, and incomplete human safety and efficacy data remain material translational challenges. Candidate inflammatory, metabolic, and endocrine biomarkers require prospective validation. These steps are necessary to determine whether PF-related molecular protection can translate into meaningful and durable clinical benefit.

## Figures and Tables

**Table 1:** Cellular mechanisms relevant to neuroinflammation-associated injury and their interpretive limits in anxiety-related pathology.

Pathway/Domain	Key Molecules or Processes	Established or Reported Findings	Interpretation for Anxiety-Related Pathology	Sources
Microglial inflammatory signaling	TLR2/4; NF-κB; ATP–P2×7	Stress-related microglial signaling can alter inflammatory responses and neuronal function.	Region- and model-dependent involvement; peripheral cytokines do not establish central microglial activation.	[3,7,11]
Mitochondrial apoptosis	BCL-2 family; BAX/BAK; cytochrome c; APAF1; caspase-9/3	Mitochondrial permeabilization can engage the apoptosome and executioner caspases.	Established cellular mechanism; its neuronal and behavioral contribution must be demonstrated in each anxiety-related model.	[39,75]
ER stress-associated apoptosis	PERK/eIF2α/ATF4; CHOP; BCL-2; redox homeostasis	Unresolved ER stress may increase apoptotic susceptibility through CHOP-associated responses.	ER stress markers do not establish completed apoptosis; rodent caspase-12 findings have species-dependent limits.	[40,44,45]
Death receptor-mediated apoptosis	Fas; TNFR1; FADD; caspase-8; BID/tBID	Receptor-specific complexes can activate caspase-8; BID links this signaling to mitochondria.	TNF-α elevation alone does not establish receptor-mediated neuronal apoptosis or a specific anxiety circuit.	[41,42]
Inflammasome-associated pyroptosis	NLRP3; ASC; caspase-1; GSDMD; IL-1β/IL-18	Inflammasome signaling can process cytokines and activate GSDMD-dependent membrane pores.	Inflammasome activation must be distinguished from demonstrated lytic death; cell identity and injury endpoints are required.	[20,21,84,96]
Apoptosis–pyroptosis crosstalk	Caspase-1; apoptotic caspases; BID; GSDMD	Caspase-1 can engage apoptotic responses without GSDMD; apoptotic cleavage can inhibit GSDMD activity.	Context-dependent cell-fate regulation; a fixed neuronal cascade in anxiety has not been established.	[43,104]

Abbreviations: TLR, Toll-like receptor; NF-κB, nuclear factor-κB; ATP, adenosine triphosphate; P2×7, purinergic P2×7 receptor; BCL-2, B-cell lymphoma 2; BAX/BAK, BCL-2-associated X protein/BCL-2 antagonist/killer; APAF1, apoptotic protease-activating factor 1; ER, endoplasmic reticulum; PERK, protein kinase R-like ER kinase; eIF2α, eukaryotic initiation factor 2α; ATF4, activating transcription factor 4; CHOP, C/EBP homologous protein; TNFR1, tumor necrosis factor receptor 1; FADD, Fas-associated death domain protein; BID/tBID, BH3-interacting domain death agonist/truncated BID; NLRP3, NLR family pyrin domain-containing 3; ASC, apoptosis-associated speck-like protein containing a CARD; GSDMD, gasdermin D; IL, interleukin. The table separates established cellular mechanisms from their model-dependent relevance to anxiety-related pathology.

**Table 2:** Selected direct PF experimental evidence across cellular, regional, neurochemical, and neuroendocrine domains.

Functional/Mechanistic Domain	Experimental Model	PF Regimen	Region/Cell Type	Measured Outcomes	Behavioral Outcome	Evidence Interpretation and Reference
Serotonergic and HPA-related measures	SPS-exposed rats	10 or 20 mg/kg, i.p.; duration not available in the accessible primary report	PFC; hippocampus; serum	Regional 5-HT/5-HIAA; circulating corticosterone, CRH, and ACTH	Improved EPM and contextual-fear outcomes; locomotion assessed	Direct PF association in a PTSD-related stress model; no proof of serotonergic mediation [150].
HPA feedback and corticosteroid receptors	SPS-exposed rats	40 mg/kg orally daily for 14 or 21 days	Hippocampus; amygdala; endocrine measures	Dexamethasone suppression; GR/MR expression; hormone measures	Stress-related behavioral assessment	Direct PF endocrine evidence; time- and region-dependent changes, not universal restoration of deficient feedback [151].
Regional neuroimmune and neuronal responses	Sleep-deprived male mice	15, 30, or 60 mg/kg/day, i.p.; duration not available in the accessible primary report	Hypothalamic PVN	Inflammatory and neuronal measures; EGFR/PI3K/Akt-related signaling	Reduced anxiety-like behavior	Direct PF anxiety-related evidence in PVN; no established hippocampus–PFC–BLA selectivity or full HPA mediation [152].
Inflammation, antioxidant responses, and synaptic measures	LPS-induced depression-like male mice; complementary BV2 experiments	Mouse: 20, 40, or 80 mg/kg/day by oral gavage for 7 days	Hippocampus; BV2 microglia	Cytokines, neuronal/synaptic measures, and Nrf2/HO-1-related responses	Improved depression-like outcomes	Direct PF cross-model support; not direct proof of anxiolysis or pathway mediation [118].
Inflammasome/pyroptosis-associated responses	CUMS-exposed mice; LPS/ATP-stimulated BV2 cells	Mouse: 40 or 80 mg/kg/day; dosing from day 28 of a 56-day CUMS protocol; oral gavage explicitly described for the 80 mg/kg inhibitor experiment	Hippocampus; BV2 microglia	SIRT1, NF-κB, NLRP3, caspase-1 and GSDMD-related measures; SIRT1-inhibitor experiments	Improved depression-like outcomes	Direct PF mechanistic support in a depression model; not an anxiety-specific mechanism [115].
Tryptophan metabolism	TDO-overexpressing cells; stress-induced depression-like mice	Mouse: oral administration; numerical dose not available in the accessible primary report	Cellular TDO system; liver	TDO-related inhibition and hepatic tryptophan metabolite measures	Depression-related outcomes assessed	Direct PF cross-model metabolic support; brain IDO1/KMO and anxiety mediation remain untested [153].
Glutamate-related responses; GABA evidence boundary	Febrile-seizure immature rats; hippocampal neuronal cultures	Rat: 100 mg/kg orally daily for 10 days from postnatal day 5; culture regimen differs	Hippocampal experimental systems	Glutamate-related calcium responses; no detected GABA production/release change under tested conditions	Anticonvulsive outcome; not an anxiety test	Direct PF cross-model evidence; no demonstrated BLA E/I normalization or generalized GABAergic effect [154].
Monoamine metabolite responses	Non-anxiety rodent experiments	Rat: 100 μg, i.c.v.; complementary mouse: 96 μg, i.c.v.	Cortical/striatal microdialysis and regional monoamine assays	Monoamine metabolite changes, including DOPAC and 5-HIAA in sampled regions	Not an anxiety-model efficacy study	Direct PF neurochemical observation with invasive delivery; no established mesolimbic dopamine normalization [155].
Oxidative-injury survival signaling	Hydrogen peroxide-exposed neural progenitor cells	NA: PF assay concentrations not available in the accessible primary report	Neural progenitor cells	Viability, apoptosis-associated measures, and PI3K/Akt-inhibitor experiments	Not applicable	Direct PF cellular mechanistic support; no anxiety-circuit or behavioral validation [10].
Direct GSDMD-target evidence	ANIT-induced cholestatic rats and HepG2 cell experiments	NA: numerical regimen not available in the accessible primary report	Liver; HepG2 cells	Target screening, CETSA/SPR binding support, and injury/pyroptosis-related endpoints	Not applicable	Direct PF target evidence in peripheral injury; CNS engagement and anxiety relevance require validation [149].

All listed studies administered PF; findings involving other drugs, PF derivatives, and combination-specific effects are excluded. i.p., intraperitoneal; i.c.v., intracerebroventricular; SPS, single prolonged stress; CUMS, chronic unpredictable mild stress; PTSD, post-traumatic stress disorder; PFC, prefrontal cortex; PVN, paraventricular nucleus; BLA, basolateral amygdala; HPA, hypothalamic–pituitary–adrenal; 5-HT, serotonin; 5-HIAA, 5-hydroxyindoleacetic acid; ACTH, adrenocorticotropic hormone; CRH, corticotropin-releasing hormone; GR/MR, glucocorticoid/mineralocorticoid receptors; EPM, elevated plus maze; TDO, tryptophan 2,3-dioxygenase; E/I, excitation/inhibition; IDO1, indoleamine 2,3-dioxygenase 1; KMO, kynurenine 3-monooxygenase; DOPAC, 3,4-dihydroxyphenylacetic acid; ANIT, α-naphthyl isothiocyanate; CETSA, cellular thermal shift assay; SPR, surface plasmon resonance. NA indicates a dose or duration that could not be extracted from the accessible primary report; it does not indicate that the information was absent from the original study. Animal regimens do not specify the concentrations used in accompanying cell experiments. Reported regimens are experimental exposures and do not define clinical doses.

## Data Availability

Data sharing is not applicable to this article as no new data were created or analyzed in this study.

## References

[1] Craske M.G., Stein M.B., Eley T.C., Milad M.R., Holmes A., Rapee R.M., Wittchen H.-U. (2017). Anxiety disorders. Nat. Rev. Dis. Primers.

[2] Weber H., Hettema J.M., Deckert J., Erhardt-Lehmann A. (2025). Genomics of Anxiety Disorders. Psychiatr. Clin. N. Am..

[3] Nie X., Kitaoka S., Tanaka K., Segi-Nishida E., Imoto Y., Ogawa A., Nakano F., Tomohiro A., Nakayama K., Taniguchi M. (2018). The Innate Immune Receptors TLR2/4 Mediate Repeated Social Defeat Stress-Induced Social Avoidance through Prefrontal Microglial Activation. Neuron.

[4] Vogelzangs N., Beekman A.T., de Jonge P., Penninx B.W. (2013). Anxiety disorders and inflammation in a large adult cohort. Transl. Psychiatry.

[5] Wang W.Y., Tan M.S., Yu J.T., Tan L. (2015). Role of pro-inflammatory cytokines released from microglia in Alzheimer’s disease. Ann. Transl. Med..

[6] Hong H., Kim B.S., Im H.I. (2016). Pathophysiological Role of Neuroinflammation in Neurodegenerative Diseases and Psychiatric Disorders. Int. Neurourol. J..

[7] Iwata M., Ota K.T., Li X.Y., Sakaue F., Li N., Dutheil S., Banasr M., Duric V., Yamanashi T., Kaneko K. (2016). Psychological Stress Activates the Inflammasome via Release of Adenosine Triphosphate and Stimulation of the Purinergic Type 2X7 Receptor. Biol. Psychiatry.

[8] Dostal C.R., Carson Sulzer M., Kelley K.W., Freund G.G., McCusker R.H. (2017). Glial and tissue-specific regulation of Kynurenine Pathway dioxygenases by acute stress of mice. Neurobiol. Stress.

[9] Tian D.D., Wang M., Liu A., Gao M.R., Qiu C., Yu W., Wang W.-J., Zhang K., Yang L., Jia Y.-Y. (2021). Antidepressant Effect of Paeoniflorin Is Through Inhibiting Pyroptosis CASP-11/GSDMD Pathway. Mol. Neurobiol..

[10] Wu Y.M., Jin R., Yang L., Zhang J., Yang Q., Guo Y.Y., Li X.-B., Liu S.-B., Luo X.-X., Zhao M.-G. (2013). Phosphatidylinositol 3 kinase/protein kinase B is responsible for the protection of paeoniflorin upon H_2_O_2_-induced neural progenitor cell injury. Neuroscience.

[11] Huang S., Dong W., Lin X., Xu K., Li K., Xiong S., Wang Z., Nie X., Bian J.-S. (2024). Disruption of the Na^+^/K^+^-ATPase-purinergic P2X7 receptor complex in microglia promotes stress-induced anxiety. Immunity.

[12] Li Q., Liu D., Pan F., Ho C.S.H., Ho R.C.M. (2019). Ethanol Exposure Induces Microglia Activation and Neuroinflammation through TLR4 Activation and SENP6 Modulation in the Adolescent Rat Hippocampus. Neural Plast..

[13] Zhenzhen Z., Fenghao L., Meina M., Rui L., Wenbo S., Qi W. (2021). Targeting HMGB1-TLR4 signaling by miR-216a-5p elevation alleviates the inflammatory behavioral hypersensitivity. Neurosci. Lett..

[14] Messeha S.S., Zarmouh N.O., Mendonca P., Kolta M.G., Soliman K.F.A. (2017). The attenuating effects of plumbagin on pro-inflammatory cytokine expression in LPS-activated BV-2 microglial cells. J. Neuroimmunol..

[15] Liu D., Wang Z., Liu S., Wang F., Zhao S., Hao A. (2011). Anti-inflammatory effects of fluoxetine in lipopolysaccharide(LPS)-stimulated microglial cells. Neuropharmacology.

[16] Hiramatsu G., Uta D., Mihara K., Andoh T., Kume T. (2021). Inhibitory effect of panaxytriol on BV-2 microglial cell activation. J. Pharmacol. Sci..

[17] Arnold P., Rickert U., Helmers A.K., Spreu J., Schneppenheim J., Lucius R. (2016). Trefoil factor 3 shows anti-inflammatory effects on activated microglia. Cell Tissue Res..

[18] Chunchai T., Pintana H., Pantiya P., Kunasol C., Thonusin C., Chattipakorn N., Chattipakorn S.C. (2026). Inhibiting Purinergic Receptor (P2X7R) Alleviates Depression- and Anxiety-Like Behaviors in Obese Rats with Immune Challenge. Acta Physiol..

[19] Paolicelli R.C., Sierra A., Stevens B., Tremblay M.E., Aguzzi A., Ajami B., Amit I., Audinat E., Bechmann I., Bennett M. (2022). Microglia states and nomenclature: A field at its crossroads. Neuron.

[20] Shi J., Zhao Y., Wang K., Shi X., Wang Y., Huang H., Zhuang Y., Cai T., Wang F., Shao F. (2015). Cleavage of GSDMD by inflammatory caspases determines pyroptotic cell death. Nature.

[21] Sborgi L., Rühl S., Mulvihill E., Pipercevic J., Heilig R., Stahlberg H., Farady C.J., Müller D.J., Broz P., Hiller S. (2016). GSDMD membrane pore formation constitutes the mechanism of pyroptotic cell death. EMBO J..

[22] Zhu J., Lee M.J., An J.H., Oh E., Chung W., Heo J.Y. (2023). ATF5 Attenuates the Secretion of Pro-Inflammatory Cytokines in Activated Microglia. Int. J. Mol. Sci..

[23] Taylor D.L., Pirianov G., Holland S., McGinnity C.J., Norman A.L., Reali C., Diemel L.T., Gveric D., Yeung D., Mehmet H. (2010). Attenuation of proliferation in oligodendrocyte precursor cells by activated microglia. J. Neurosci. Res..

[24] Yan L., Yang J., Yu M., Lu Y., Huang L., Wang J., Lu X., Jin C., Wu S., Cai Y. (2019). Lanthanum chloride induces neuron damage by activating the nuclear factor-kappa B signaling pathway in activated microglia. Metallomics.

[25] Li X., Huang H., Li Y., Feng Y., Wang J., Luo S., Chen Y., Zhang Y., Yan G., Nan L. (2024). Gualou Guizhi Granule inhibits microglia-mediated neuroinflammation to protect against neuronal apoptosis in vitro and in vivo. Front. Immunol..

[26] Zhang Y.P., Li Y.Y., Zhang C., Li Y.J., Liu B.P., Zhang Y., Lin J.-D., Song C. (2022). Interleukin-10 Attenuates Behavioral, Immune and Neurotrophin Changes Induced by Chronic Central Administration of Interleukin-1β in Rats. Neuroimmunomodulation.

[27] Zheng Z.H., Tu J.L., Li X.H., Hua Q., Liu W.Z., Liu Y., Pan B.-X., Hu P., Zhang W.-H. (2021). Neuroinflammation induces anxiety- and depressive-like behavior by modulating neuronal plasticity in the basolateral amygdala. Brain Behav. Immun..

[28] Hill M.N., Kumar S.A., Filipski S.B., Iverson M., Stuhr K.L., Keith J.M., Cravatt B.F., Hillard C.J., Chattarji S., McEwen B.S. (2013). Disruption of fatty acid amide hydrolase activity prevents the effects of chronic stress on anxiety and amygdalar microstructure. Mol. Psychiatry.

[29] Koo J.W., Duman R.S. (2008). IL-1beta is an essential mediator of the antineurogenic and anhedonic effects of stress. Proc. Natl. Acad. Sci. USA.

[30] Anacker C., Luna V.M., Stevens G.S., Millette A., Shores R., Jimenez J.C., Chen B., Hen R. (2018). Hippocampal neurogenesis confers stress resilience by inhibiting the ventral dentate gyrus. Nature.

[31] Snyder J.S., Soumier A., Brewer M., Pickel J., Cameron H.A. (2011). Adult hippocampal neurogenesis buffers stress responses and depressive behaviour. Nature.

[32] Matsuura S., Nishimoto Y., Endo A., Shiraki H., Suzuki K., Segi-Nishida E. (2023). Hippocampal Inflammation and Gene Expression Changes in Peripheral Lipopolysaccharide Challenged Mice Showing Sickness and Anxiety-Like Behaviors. Biol. Pharm. Bull..

[33] Shentu Y., Tian Q., Yang J., Liu X., Han Y., Yang D., Zhang N., Fan X., Wang P., Ma J. (2021). Upregulation of KDM6B contributes to lipopolysaccharide-induced anxiety-like behavior via modulation of VGLL4 in mice. Behav. Brain Res..

[34] Woo H., Hong C.J., Jung S., Choe S., Yu S.W. (2018). Chronic restraint stress induces hippocampal memory deficits by impairing insulin signaling. Mol. Brain.

[35] Adhikari A., Lerner T.N., Finkelstein J., Pak S., Jennings J.H., Davidson T.J., Ferenczi E., Gunaydin L.A., Mirzabekov J.J., Ye L. (2015). Basomedial amygdala mediates top-down control of anxiety and fear. Nature.

[36] Dehdar K., Mooziri M., Samii Moghaddam A., Salimi M., Nazari M., Dehghan S., Jamaati H., Salimi A., Raoufy M.R. (2023). Corticosteroid treatment attenuates anxiety and mPFC-amygdala circuit dysfunction in allergic asthma. Life Sci..

[37] Yan L., Wang M., Yang F., Wang Y., Wang S., So K.F., Zhang L. (2023). Physical exercise mediates a cortical FMRP-mTOR pathway to improve resilience against chronic stress in adolescent mice. Transl. Psychiatry.

[38] Elliott E., Manashirov S., Zwang R., Gil S., Tsoory M., Shemesh Y., Chen A. (2016). Dnmt3a in the Medial Prefrontal Cortex Regulates Anxiety-Like Behavior in Adult Mice. J. Neurosci..

[39] Li P., Nijhawan D., Budihardjo I., Srinivasula S.M., Ahmad M., Alnemri E.S., Wang X. (1997). Cytochrome c and dATP-dependent formation of Apaf-1/caspase-9 complex initiates an apoptotic protease cascade. Cell.

[40] McCullough K.D., Martindale J.L., Klotz L.O., Aw T.Y., Holbrook N.J. (2001). Gadd153 sensitizes cells to endoplasmic reticulum stress by down-regulating Bcl2 and perturbing the cellular redox state. Mol. Cell. Biol..

[41] Micheau O., Tschopp J. (2003). Induction of TNF receptor I-mediated apoptosis via two sequential signaling complexes. Cell.

[42] Luo X., Budihardjo I., Zou H., Slaughter C., Wang X. (1998). Bid, a Bcl2 interacting protein, mediates cytochrome c release from mitochondria in response to activation of cell surface death receptors. Cell.

[43] Tsuchiya K., Nakajima S., Hosojima S., Thi Nguyen D., Hattori T., Manh Le T., Hori O., Mahib M.R., Yamaguchi Y., Miura M. (2019). Caspase-1 initiates apoptosis in the absence of gasdermin D. Nat. Commun..

[44] Nakagawa T., Zhu H., Morishima N., Li E., Xu J., Yankner B.A., Yuan J. (2000). Caspase-12 mediates endoplasmic-reticulum-specific apoptosis and cytotoxicity by amyloid-beta. Nature.

[45] Saleh M., Vaillancourt J.P., Graham R.K., Huyck M., Srinivasula S.M., Alnemri E.S., Steinberg M.H., Nolan V., Baldwin C.T., Hotchkiss R.S. (2004). Differential modulation of endotoxin responsiveness by human caspase-12 polymorphisms. Nature.

[46] Hsieh H.L., Yang C.M. (2013). Role of redox signaling in neuroinflammation and neurodegenerative diseases. BioMed Res. Int..

[47] Okuda S., Nishiyama N., Saito H., Katsuki H. (1998). 3-Hydroxykynurenine, an endogenous oxidative stress generator, causes neuronal cell death with apoptotic features and region selectivity. J. Neurochem..

[48] Nakai M., Qin Z.H., Wang Y., Chase T.N. (1999). Free radical scavenger OPC-14117 attenuates quinolinic acid-induced NF-kappaB activation and apoptosis in rat striatum. Brain Res. Mol. Brain Res..

[49] Lin C.C., Hsieh H.L., Shih R.H., Chi P.L., Cheng S.E., Chen J.C., Yang C.-M. (2012). NADPH oxidase 2-derived reactive oxygen species signal contributes to bradykinin-induced matrix metalloproteinase-9 expression and cell migration in brain astrocytes. Cell Commun. Signal..

[50] Yang C.M., Hsieh H.L., Yu P.H., Lin C.C., Liu S.W. (2015). IL-1β Induces MMP-9-Dependent Brain Astrocytic Migration via Transactivation of PDGF Receptor/NADPH Oxidase 2-Derived Reactive Oxygen Species Signals. Mol. Neurobiol..

[51] Xu T., Sun L., Shen X., Chen Y., Yin Y., Zhang J., Huang D., Li W., Li W. (2019). NADPH oxidase 2-mediated NLRP1 inflammasome activation involves in neuronal senescence in hippocampal neurons in vitro. Int. Immunopharmacol..

[52] Villar-Vesga J., De Feo D., Clément P., Bugada V., Meuffels E., Van Hove H., Bijnen M., King J., Grundschober S., Ulutekin C. (2026). Monocyte-derived macrophages drive neurological tissue damage through mitochondrial reactive oxygen species. Sci. Immunol..

[53] Chen J., Yin W., Tu Y., Wang S., Yang X., Chen Q., Zhang X., Han Y., Pi R. (2017). L-F001, a novel multifunctional ROCK inhibitor, suppresses neuroinflammation in vitro and in vivo: Involvement of NF-κB inhibition and Nrf2 pathway activation. Eur. J. Pharmacol..

[54] Zhao X., Huang X., Yang C., Jiang Y., Zhou W., Zheng W. (2022). Artemisinin Attenuates Amyloid-Induced Brain Inflammation and Memory Impairments by Modulating TLR4/NF-κB Signaling. Int. J. Mol. Sci..

[55] Lin H.W., Chen T.C., Wang I., Yeh J.H., Tsou S.C., Sun L., Chung S.-C., Chuang C.-J., Chang Y.-Y. (2025). Tetrahydrocurcumin Protects Microglial Cells Against Pseudomonas aeruginosa Lipopolysaccharide-Induced Reactive Oxygen Species Production and Cathepsin B to Activate NLRP3 Inflammasome-Mediated Pyroptosis Via the HO-1 and p38/JNK Pathway. Int. J. Med. Sci..

[56] Jabri M.A., Rtibi K., Sebai H. (2022). Chamomile decoction mitigates high fat diet-induced anxiety-like behavior, neuroinflammation and cerebral ROS overload. Nutr. Neurosci..

[57] Wang Y., Pu M., Wang X. (2026). Netrin-3 rectifies diabetes-induced cognitive impairment by counteracting hippocampal oxidative stress and NLRP3 inflammasome activation. Neuropeptides.

[58] Zhang J.B., Lu G.D., Sun D.N., Ding P., Chen Y.K., Zhou Y.Y., Chen Y.-T., Cai M., Ryu J.H., Wang P. (2025). Pellitorine protects chronic restraint stress-induced cognitive deficits via inhibiting neural inflammation and ferroptosis. Int. Immunopharmacol..

[59] Robinson C.M., Shirey K.A., Carlin J.M. (2003). Synergistic transcriptional activation of indoleamine dioxygenase by IFN-gamma and tumor necrosis factor-alpha. J. Interferon Cytokine Res..

[60] Guillemin G.J., Kerr S.J., Smythe G.A., Smith D.G., Kapoor V., Armati P.J., Croitoru J., Brew B.J. (2001). Kynurenine pathway metabolism in human astrocytes: A paradox for neuronal protection. J. Neurochem..

[61] Kessler M., Terramani T., Lynch G., Baudry M. (1989). A glycine site associated with N-methyl-D-aspartic acid receptors: Characterization and identification of a new class of antagonists. J. Neurochem..

[62] Deng Y., Zhou M., Wang J., Yao J., Yu J., Liu W., Wu L., Wang J., Gao R. (2021). Involvement of the microbiota-gut-brain axis in chronic restraint stress: Disturbances of the kynurenine metabolic pathway in both the gut and brain. Gut Microbes.

[63] Butler M.I., Long-Smith C., Moloney G.M., Morkl S., O’Mahony S.M., Cryan J.F., Clarke G., Dinan T.G. (2022). The immune-kynurenine pathway in social anxiety disorder. Brain Behav. Immun..

[64] Boyle M.P., Brewer J.A., Funatsu M., Wozniak D.F., Tsien J.Z., Izumi Y., Muglia L.J. (2005). Acquired deficit of forebrain glucocorticoid receptor produces depression-like changes in adrenal axis regulation and behavior. Proc. Natl. Acad. Sci. USA.

[65] Frank M.G., Thompson B.M., Watkins L.R., Maier S.F. (2012). Glucocorticoids mediate stress-induced priming of microglial pro-inflammatory responses. Brain Behav. Immun..

[66] Cohen S., Janicki-Deverts D., Doyle W.J., Miller G.E., Frank E., Rabin B.S., Turner R.B. (2012). Chronic stress, glucocorticoid receptor resistance, inflammation, and disease risk. Proc. Natl. Acad. Sci. USA.

[67] McKim D.B., Weber M.D., Niraula A., Sawicki C.M., Liu X., Jarrett B.L., Ramirez-Chan K., Wang Y., Roeth R.M., Sucaldito A.D. (2018). Microglial recruitment of IL-1β-producing monocytes to brain endothelium causes stress-induced anxiety. Mol. Psychiatry.

[68] Menard C., Pfau M.L., Hodes G.E., Kana V., Wang V.X., Bouchard S., Takahashi A., Flanigan M.E., Aleyasin H., LeClair K.B. (2017). Social stress induces neurovascular pathology promoting depression. Nat. Neurosci..

[69] Wu X., Ding Z., Fan T., Wang K., Li S., Zhao J., Zhu W. (2022). Childhood social isolation causes anxiety-like behaviors via the damage of blood-brain barrier in amygdala in female mice. Front. Cell Dev. Biol..

[70] Bonorino K.C., Iria Kraus S., Henrique Cardoso Martins G., Jorge Probst J., Petry Moeke D.M., Henrique Dos Santos Sumar A., Casal Y.R., Oliveira F.R.M.B., Sordi R., Assreuy J. (2024). Lung-brain crosstalk: Behavioral disorders and neuroinflammation in septic survivor mice. Brain Behav. Immun. Health.

[71] Li S., Qian Q., Xie Y., Wu Z., Yang H., Yin Y., Cui Y., Li X. (2024). Ameliorated Effects of Fucoidan on Dextran Sulfate Sodium-Induced Ulcerative Colitis and Accompanying Anxiety and Depressive Behaviors in Aged C57BL/6 Mice. J. Agric. Food Chem..

[72] Wang X., Wu H., Miller A.H. (2004). Interleukin 1alpha (IL-1alpha) induced activation of p38 mitogen-activated protein kinase inhibits glucocorticoid receptor function. Mol. Psychiatry.

[73] Zorn J.V., Schür R.R., Boks M.P., Kahn R.S., Joëls M., Vinkers C.H. (2017). Cortisol stress reactivity across psychiatric disorders: A systematic review and meta-analysis. Psychoneuroendocrinology.

[74] Barik J., Marti F., Morel C., Fernandez S.P., Lanteri C., Godeheu G., Tassin J.-P., Mombereau C., Faure P., Tronche F. (2013). Chronic stress triggers social aversion via glucocorticoid receptor in dopaminoceptive neurons. Science.

[75] Wei M.C., Zong W.X., Cheng E.H., Lindsten T., Panoutsakopoulou V., Ross A.J., Roth K.A., MacGregor G.R., Thompson C.B., Korsmeyer S.J. (2001). Proapoptotic BAX and BAK: A requisite gateway to mitochondrial dysfunction and death. Science.

[76] Liao D., Xiang D., Dang R., Xu P., Wang J., Han W., Fu Y., Yao D., Cao L., Jiang P. (2018). Neuroprotective Effects of dl-3-n-Butylphthalide against Doxorubicin-Induced Neuroinflammation, Oxidative Stress, Endoplasmic Reticulum Stress, and Behavioral Changes. Oxidative Med. Cell. Longev..

[77] Yang Y., Wang J., Wang S., Chen Y., Li Z., Zhang H., Zhou P., Li W., Ren D., Sun H. (2025). Gypenoside XVII regulates apoptosis and fear extinction deficits mediated by post-traumatic stress disorder through the PERK/CHOP signaling pathway. Eur. J. Pharmacol..

[78] Fluhr H., Spratte J., Bredow M., Heidrich S., Zygmunt M. (2013). Constitutive activity of Erk1/2 and NF-κB protects human endometrial stromal cells from death receptor-mediated apoptosis. Reprod. Biol..

[79] Chacko A.D., Liberante F., Paul I., Longley D.B., Fennell D.A. (2010). Voltage dependent anion channel-1 regulates death receptor mediated apoptosis by enabling cleavage of caspase-8. BMC Cancer.

[80] Zhu S., Li T., Tan J., Yan X., Zhang D., Zheng C., Chen Y., Xiang Z., Cui H. (2012). Bax is essential for death receptor-mediated apoptosis in human colon cancer cells. Cancer Biother. Radiopharm..

[81] Song N., Liu Z.S., Xue W., Bai Z.F., Wang Q.Y., Dai J., Liu X., Huang Y.-J., Cai H., Zhan X.-Y. (2017). NLRP3 Phosphorylation Is an Essential Priming Event for Inflammasome Activation. Mol. Cell.

[82] Saller B.S., Neuwirt E., Groß O. (2023). Methods to Activate the NLRP3 Inflammasome. Methods in Molecular Biology.

[83] Ren G.M., Li J., Zhang X.C., Wang Y., Xiao Y., Zhang X.Y., Liu X., Zhang W., Ma W.-B., Zhang J. (2021). Pharmacological targeting of NLRP3 deubiquitination for treatment of NLRP3-associated inflammatory diseases. Sci. Immunol..

[84] Lei Y., Chen C.J., Yan X.X., Li Z., Deng X.H. (2017). Early-life lipopolysaccharide exposure potentiates forebrain expression of NLRP3 inflammasome proteins and anxiety-like behavior in adolescent rats. Brain Res..

[85] Zhai Y., Meng X., Ye T., Xie W., Sun G., Sun X. (2018). Inhibiting the NLRP3 Inflammasome Activation with MCC950 Ameliorates Diabetic Encephalopathy in db/db Mice. Molecules.

[86] Li Y., Song W., Tong Y., Zhang X., Zhao J., Gao X., Yong J., Wang H. (2021). Isoliquiritin ameliorates depression by suppressing NLRP3-mediated pyroptosis via miRNA-27a/SYK/NF-κB axis. J. Neuroinflamm..

[87] Sahin C., Albayrak O., Akdeniz T.F., Akbulut Z., Yanikkaya Demirel G., Aricioglu F. (2016). Agmatine Reverses Sub-chronic Stress induced Nod-like Receptor Protein 3 (NLRP3) Activation and Cytokine Response in Rats. Basic Clin. Pharmacol. Toxicol..

[88] Mei T., Ma L., Kong F. (2023). Sodium aescinate improve behavioral performance by inhibiting dorsal raphe nucleus NLRP3 inflammasome in Post-traumatic stress disorder Rat Model. Biochem. Biophys. Res. Commun..

[89] Chen L., Qing W., Yi Z., Lin G., Peng Q., Zhou F. (2021). NU9056, a KAT 5 Inhibitor, Treatment Alleviates Brain Dysfunction by Inhibiting NLRP3 Inflammasome Activation, Affecting Gut Microbiota, and Derived Metabolites in LPS-Treated Mice. Front. Nutr..

[90] Yu X., Yu X., Yang Y., Cheng W., Shi M., Chen L., Zhang X., Xu Y. (2025). Probiotic Bifico Ameliorates Depression- and Anxiety-Like Behaviors Induced by Estrogen Deficiency via NLRP3 Inflammasome Inhibition. J. Inflamm. Res..

[91] Ma Q., Chen S., Hu Q., Feng H., Zhang J.H., Tang J. (2014). NLRP3 inflammasome contributes to inflammation after intracerebral hemorrhage. Ann. Neurol..

[92] Shi Y., Hu Y., Gan Y., Mi Z., Luo S., Lei J., Fang Q., Li H. (2025). Tabersonine ameliorates depressive-like behavior by inhibiting NLRP3 inflammasome activation in a mouse model. Neuropharmacology.

[93] Ruan J. (2019). Structural Insight of Gasdermin Family Driving Pyroptotic Cell Death. Advances in Experimental Medicine and Biology.

[94] Liu Z., Li S., Wang C., Vidmar K.J., Bracey S., Li L., Willard B., Miyagi M., Lan T., Dickinson B.C. (2024). Palmitoylation at a conserved cysteine residue facilitates gasdermin D-mediated pyroptosis and cytokine release. Proc. Natl. Acad. Sci. USA.

[95] Shi Y., Yang Y., Xu W., Shi D., Xu W., Fu X., Lv Q., Xia J., Shi F. (2022). E3 ubiquitin ligase SYVN1 is a key positive regulator for GSDMD-mediated pyroptosis. Cell Death Dis..

[96] Lei W.X., Zhang L., Chen J.L., Zheng G.H., Guo L.N., Jiang T., Yin Z.-Y., Ying M., Yu Q.-M., Wang N. (2024). The role and mechanism of miR-425-3p regulating neuronal pyroptosis-mediated inorganic arsenic-induced generalized anxiety disorder. Ecotoxicol. Environ. Saf..

[97] Wu D., Li X., Xing Y., Li Y., Li Y., Li C., Wang Y., Li H., Liu J., Zhang W. (2025). The role of IL-18BP in alleviating anxiety-like behaviors after traumatic brain injury in rats by modulating astrocytic pyroptosis in amygdala. Brain Inj..

[98] Song T., Chen Y., Li C., Yao Y., Ma S., Shang Y., Cheng J. (2024). Identification of Molecular Correlations of GSDMD with Pyroptosis in Alzheimer’s Disease. Comb. Chem. High Throughput Screen..

[99] Miao H.T., Wang J., Shao J.J., Song R.X., Li W.G., Sun J.K., Jia S.-Y., Zhang D.-X., Li X.-M., Zhao J.-Y. (2025). Astrocytic NLRP3 cKO mitigates depression-like behaviors induced by mild TBI in mice. Neurobiol. Dis..

[100] Zhang L.M., Zhang D.X., Song R.X., Lv J.M., Wang L.Y., Wu Z.Y., Miao H.-T., Zhou Y.-B., Zhang W., Xin Y. (2023). IL-18BP Alleviates Anxiety-Like Behavior Induced by Traumatic Stress via Inhibition of the IL-18R-NLRP3 Signaling Pathway in a Mouse Model of Hemorrhagic Shock and Resuscitation. Mol. Neurobiol..

[101] Chen L.P., Gui X.D., Tian W.D., Kan H.M., Huang J.Z., Ji F.H. (2024). Botulinum toxin type A-targeted SPP1 contributes to neuropathic pain by the activation of microglia pyroptosis. World J. Psychiatry.

[102] An D., Xu W., Ge Y., Ge Y., Zhang L., Zhu Y., Zhang Z., Fan J., Gao R., Jiang L. (2023). Protection of Oxygen Glucose Deprivation-Induced Human Brain Vascular Pericyte Injury: Beneficial Effects of Bellidifolin in Cellular Pyroptosis. Neurochem. Res..

[103] Chuang H.C., Nichols E.K., Rauch I., Chang W.C., Lin P.M., Misra R., Kitaoka M., Vance R.E., Saijo K. (2021). Lytic Cell Death in Specific Microglial Subsets Is Required for Preventing Atypical Behavior in Mice. eNeuro.

[104] Taabazuing C.Y., Okondo M.C., Bachovchin D.A. (2017). Pyroptosis and Apoptosis Pathways Engage in Bidirectional Crosstalk in Monocytes and Macrophages. Cell Chem. Biol..

[105] Cui Y., Hu Z., Wang L., Zhu B., Deng L., Zhang H., Wang X. (2024). DL-3-n-Butylphthalide Ameliorates Post-stroke Emotional Disorders by Suppressing Neuroinflammation and PANoptosis. Neurochem. Res..

[106] Wright S.S., Kumari P., Fraile-Ágreda V., Wang C., Shivcharan S., Kappelhoff S., Margheritis E.G., Matz A., Vasudevan S.O., Rubio I. (2025). Transplantation of gasdermin pores by extracellular vesicles propagates pyroptosis to bystander cells. Cell.

[107] Sharma B.R., Kanneganti T.D. (2025). Extracellular vesicles transport gasdermin pores, amplifying inflammatory cell death. Trends Immunol..

[108] Xu W., Zhao Y., Qin Y., Ge B., Gong W., Wu Y., Li X., Zhao Y., Xu P., Xue M. (2016). Enhancement of Exposure and Reduction of Elimination for Paeoniflorin or Albiflorin via Co-Administration with Total Peony Glucosides and Hypoxic Pharmacokinetics Comparison. Molecules.

[109] Jiao F., Varghese K., Wang S., Liu Y., Yu H., Booz G.W., Roman R.J., Liu R., Fan F. (2021). Recent Insights Into the Protective Mechanisms of Paeoniflorin in Neurological, Cardiovascular, and Renal Diseases. J. Cardiovasc. Pharmacol..

[110] Takeda S., Isono T., Wakui Y., Matsuzaki Y., Sasaki H., Amagaya S., Maruno M. (1995). Absorption and excretion of paeoniflorin in rats. J. Pharm. Pharmacol..

[111] Yu J.B., Zhao Z.X., Peng R., Pan L.B., Fu J., Ma S.R., Han P., Cong L., Zhang Z.-W., Sun L.-X. (2019). Gut Microbiota-Based Pharmacokinetics and the Antidepressant Mechanism of Paeoniflorin. Front. Pharmacol..

[112] Qian J., Cheng W., Zhang C., Hong L., Chen W., Li G. (2019). Preparation, physicochemical characterization and pharmacokinetics of paeoniflorin-phospholipid complex. Biomed. Mater. Eng..

[113] Hu P.Y., Liu D., Zheng Q., Wu Q., Tang Y., Yang M. (2016). Elucidation of Transport Mechanism of Paeoniflorin and the Influence of Ligustilide, Senkyunolide I and Senkyunolide A on Paeoniflorin Transport through Mdck-Mdr1 Cells as Blood-Brain Barrier in Vitro Model. Molecules.

[114] Yang X., Guo Q., Dong Y., Wang H., Dang M., Sun Y., Gao Y., Bai D. (2025). Glycyrrhiza Protein-Based Nanoparticles Enhance the Oral Bioavailability and Analgesic Efficacy of Paeoniflorin. Int. J. Nanomed..

[115] Wang X., Su L., Liu S., He Z., Li J., Zong Y., Chen W., Du R. (2024). Paeoniflorin Inhibits the Activation of Microglia and Alleviates Depressive Behavior by Regulating SIRT1-NF-kB-NLRP3/Pyroptosis Pathway. Int. J. Mol. Sci..

[116] Chen Q., Liu Y., Zhang Y., Jiang X., Zhang Y., Asakawa T. (2020). An In Vitro Verification of the Effects of Paeoniflorin on Lipopolysaccharide-Exposed Microglia. Evid.-Based Complement. Altern. Med..

[117] Nam K.N., Yae C.G., Hong J.W., Cho D.H., Lee J.H., Lee E.H. (2013). Paeoniflorin, a monoterpene glycoside, attenuates lipopolysaccharide-induced neuronal injury and brain microglial inflammatory response. Biotechnol. Lett..

[118] Hu Z., Wang X., Shi T., Yang L., Zhang B., Shang B., He R., Yi S., He J., Hu J. (2025). Paeoniflorin Alleviates Lipopolysaccharide-Induced Neuroinflammation and Depression Through the Keap1/Nrf2/HO-1 Signaling Pathway. Antioxidants.

[119] Liu H., Wang J., Wang J., Wang P., Xue Y. (2015). Paeoniflorin attenuates Aβ1-42-induced inflammation and chemotaxis of microglia in vitro and inhibits NF-κB- and VEGF/Flt-1 signaling pathways. Brain Res..

[120] Choi J.W., Im J.H., Balakrishnan R. (2025). Paeoniflorin exercise-mimetic potential regulates the Nrf2/HO-1/BDNF/CREB and APP/BACE-1/NF-κB/MAPK signaling pathways to reduce cognitive impairments and neuroinflammation in amnesic mouse model. Biomed. Pharmacother..

[121] Sun Y., Wang G., Li S., Jiang Y., Liu Y., Gao Y., Yuan Y., Nie H. (2025). Paeoniflorin Directly Targets ENO1 to Inhibit M1 Polarization of Microglia/Macrophages and Ameliorates EAE Disease. Int. J. Mol. Sci..

[122] Zhou X., Chen X., Cheng X., Lin L., Quan S., Li S., Zhan R., Wu Q., Liu S. (2023). Paeoniflorin, ferulic acid, and atractylenolide III improved LPS-induced neuroinflammation of BV2 microglia cells by enhancing autophagy. J. Pharmacol. Sci..

[123] Zheng Z.H., Lin X.C., Lu Y., Cao S.R., Liu X.K., Lin D., Yang F.-H., Zhang Y.-B., Tu J.-L., Pan B.-X. (2023). Harmine exerts anxiolytic effects by regulating neuroinflammation and neuronal plasticity in the basolateral amygdala. Int. Immunopharmacol..

[124] Bawadood A.S., Al-Abbasi F.A., Alghamdi A.M., Alqurashi M.M., Sheikh R.A., Alzarea S.I., Sayyed N., Kazmi I. (2024). Fustin alleviates lipopolysaccharide-induced anxiety-depression-like performances by modulation of oxidative stress/neuroinflammatory markers/ NF-κB/caspase-3/BDNF expression in rodents. Eur. Rev. Med. Pharmacol. Sci..

[125] Shentu Y., Chen M., Wang H., Du X., Zhang W., Xie G., Zhou S., Ding L., Zhu Y., Zhu M. (2024). Hydrogen sulfide ameliorates lipopolysaccharide-induced anxiety-like behavior by inhibiting checkpoint kinase 1 activation in the hippocampus of mice. Exp. Neurol..

[126] Wang X., Weng G., Gao Y., Wang Y., Zhang C. (2025). Quercetin ameliorates chronic restraint stress- and LPS-induced anxiety-like behaviors by modulating neuroinflammation in the lateral hypothalamus. Psychopharmacology.

[127] Wang G., Tang Q., Wang D. (2026). Exploring the mechanism by which puerarin inhibits neuroinflammation and alleviates lipopolysaccharide-induced anxiety-like behavior in mice via modulating gut microbiota based on the brain-gut axis theory. Brain Res. Bull..

[128] Wang L., Li M., Dong Y., Wang J., Qin S., Li L., Li B., Wang B., Cao H. (2025). Magnoflorine alleviates colitis-induced anxiety-like behaviors by regulating gut microbiota and microglia-mediated neuroinflammation. Microbiome.

[129] Yang X., Yao W., Shi H., Liu H., Li Y., Gao Y., Liu R., Xu L. (2016). Paeoniflorin protects Schwann cells against high glucose induced oxidative injury by activating Nrf2/ARE pathway and inhibiting apoptosis. J. Ethnopharmacol..

[130] Lin J., Xu F., Wang G., Kong L., Luo Q., Lv Y., Liu J., Wei Y., Li L., Zhang H. (2016). Paeoniflorin Attenuated Oxidative Stress in Rat COPD Model Induced by Cigarette Smoke. Evid.-Based Complement. Altern. Med..

[131] Yuan J., Lu Y., Wang H., Feng Y., Jiang S., Gao X.H., Qi R., Wu Y., Chen H.-D. (2020). Paeoniflorin Resists H_2_O_2_-Induced Oxidative Stress in Melanocytes by JNK/Nrf2/HO-1 Pathway. Front. Pharmacol..

[132] Li L., Wang H., Zhao S., Zhao Y., Chen Y., Zhang J., Wang C., Sun N., Fan H. (2022). Paeoniflorin ameliorates lipopolysaccharide-induced acute liver injury by inhibiting oxidative stress and inflammation via SIRT1/FOXO1a/SOD2 signaling in rats. Phytother. Res..

[133] Yang F., Li Y., Sheng X., Liu Y. (2021). Paeoniflorin treatment regulates TLR4/NF-κB signaling, reduces cerebral oxidative stress and improves white matter integrity in neonatal hypoxic brain injury. Korean J. Physiol. Pharmacol..

[134] Yu X., Fan Z., Han Y., Zhang D., Xu L., Wang M., Yang Q., Li H., Zhou M., Zhang L. (2018). Paeoniflorin reduces neomycin-induced ototoxicity in hair cells by suppression of reactive oxygen species generation and extracellularly regulated kinase signalization. Toxicol. Lett..

[135] Yu X., Man R., Li Y., Yang Q., Li H., Yang H., Bai X., Yin H., Li J., Wang H. (2019). Paeoniflorin protects spiral ganglion neurons from cisplatin-induced ototoxicity: Possible relation to PINK1/BAD pathway. J. Cell. Mol. Med..

[136] Chen X., Sun T., Qi Y., Zhu B., Li L., Yu J., Ding Z., Zhou F. (2025). Paeoniflorin ameliorates reperfusion injury in H9C2 cells through SIRT1-PINK1/parkin-mediated mitochondrial autophagy. Mol. Immunol..

[137] Yang X., Li X., Zhu Y., Gao Y., Xu L. (2022). Paeoniflorin Upregulates Mitochondrial Thioredoxin of Schwann Cells to Improve Diabetic Peripheral Neuropathy Indicated by 4D Label-Free Quantitative Proteomics. Oxidative Med. Cell. Longev..

[138] Jiang Z., Chen W., Yan X., Bi L., Guo S., Zhan Z. (2014). Paeoniflorin protects cells from GalN/TNF-α-induced apoptosis via ER stress and mitochondria-dependent pathways in human L02 hepatocytes. Acta Biochim. Biophys. Sin..

[139] Zhu X., Wang K., Zhou F., Zhu L. (2018). Paeoniflorin attenuates atRAL-induced oxidative stress, mitochondrial dysfunction and endoplasmic reticulum stress in retinal pigment epithelial cells via triggering Ca^2+^/CaMKII-dependent activation of AMPK. Arch. Pharmacal Res..

[140] Chen J., Zhang M., Zhu M., Gu J., Song J., Cui L., Liu D., Ning Q., Jia X., Feng L. (2018). Paeoniflorin prevents endoplasmic reticulum stress-associated inflammation in lipopolysaccharide-stimulated human umbilical vein endothelial cells via the IRE1α/NF-κB signaling pathway. Food Funct..

[141] Su L., Guo P., Guo X., He Z., Zhao Y., Zong Y., Li J., Chen W., Du R. (2024). Paeoniflorin alleviates depression by inhibiting the activation of NLRP3 inflammasome via promoting mitochondrial autophagy. Chin. J. Nat. Med..

[142] Liu P., Cheng J., Ma S., Zhou J. (2020). Paeoniflorin attenuates chronic constriction injury-induced neuropathic pain by suppressing spinal NLRP3 inflammasome activation. Inflammopharmacology.

[143] Jiang T., Liu X., Wang S., Chen Y., Wang Y., Li X., Yao G. (2025). Paeoniflorin alleviated experimental Sjögren’s syndrome by inhibiting NLRP3 inflammasome activation of submandibular gland cells via activating Nrf2/HO-1 pathway. Free Radic. Biol. Med..

[144] Zhang J., Zhang W., Fan B., Yang Z., Tian Z., Wang C., Shang L., Zhang Z. (2026). Paeoniflorin Alleviates Pulmonary Arterial Hypertension by Suppressing TRIM24-Mediated SIRT1 Ubiquitination and NLRP3 Inflammasome Activation. Chem. Biol. Drug Des..

[145] Wang A., Gong Y., Pei Z., Jiang L., Xia L., Wu Y. (2022). Paeoniflorin ameliorates diabetic liver injury by targeting the TXNIP-mediated NLRP3 inflammasome in db/db mice. Int. Immunopharmacol..

[146] Guo N., Geng Q., Wang Y., Sun Y., Xu H., Wu S., Li Y., Leng R., Qin W., Chen S. (2025). Paeoniflorin Alleviates Metabolic Dysfunction-Associated Fatty Liver Disease by Targeting STING-Mediated Pyroptosis via Inhibiting the NLRP3 Inflammasome. Am. J. Chin. Med..

[147] Zhang H., Yang X., Wang Y., Xiao W., Zhao Y., Wu F., Liu H., Ren S., Liu Y., Lin Y. (2026). Paeoniflorin suppresses cardiomyocyte pyroptosis and ameliorates diabetic cardiomyopathy by AMPK/Nrf2/NLRP3 pathway. Int. Immunopharmacol..

[148] Yu J., Zhang R., Quan Y., Sun D., Huang C., Xu J., Li X., Liu L. (2026). Paeoniflorin Ameliorates Alcohol-Induced Depression via Modulating the Gut-Brain Axis and Inhibiting the NF-κB/NLRP3 Inflammasome Pathway in Mice. Phytother. Res..

[149] Ma X., Zhang W., Chen Y., Hu Q., Wang Z., Jiang T., Zeng Y., Efferth T. (2024). Paeoniflorin inhibited GSDMD to alleviate ANIT-induced cholestasis via pyroptosis signaling pathway. Phytomedicine.

[150] Qiu Z.K., He J.L., Liu X., Zeng J., Xiao W., Fan Q.H., Chai X.-M., Ye W.-H., Chen J.-S. (2018). Anxiolytic-like effects of paeoniflorin in an animal model of post traumatic stress disorder. Metab. Brain Dis..

[151] Chen J., Ye W., Li L., Su J., Huang Y., Liu L., Wu L., Yan C. (2020). Paeoniflorin regulates the hypothalamic-pituitary-adrenal axis negative feedback in a rat model of post-traumatic stress disorder. Iran. J. Basic. Med. Sci..

[152] Gong H., Wu Z., Liu R., Zhang Y., Song D., Wang M., Li X., Wu Y., Zhao M., Feng B. (2026). Paeoniflorin alleviates anxiety-like behaviors in sleep-deprived male mice by suppressing inflammation of the paraventricular nucleus of the hypothalamus. Phytomedicine.

[153] Liang X., Su T., Wu P., Dai Y., Chen Y., Wang Q., Cao C., Chen F., Wang Q., Wang S. (2023). Identification of paeoniflorin from Paeonia lactiflora pall. As an inhibitor of tryptophan 2,3-dioxygenase and assessment of its pharmacological effects on depressive mice. J. Ethnopharmacol..

[154] Hino H., Takahashi H., Suzuki Y., Tanaka J., Ishii E., Fukuda M. (2012). Anticonvulsive effect of paeoniflorin on experimental febrile seizures in immature rats: Possible application for febrile seizures in children. PLoS ONE.

[155] Lin Y.T., Huang W.S., Tsai H.Y., Lee M.M., Chen Y.F. (2019). In vivo microdialysis and in vitro HPLC analysis of the impact of paeoniflorin on the monoamine levels and their metabolites in the rodent brain. Biomedicine.

[156] Guo Y., Wei F., Lv Y., Wu H., Li Y., Tang R., Zhao M., Ge W., Du W. (2024). Spinosin improves anxiety disorders in mice with chronic restraint stress via the ERK1/2-CREB-BDNF pathway. Int. Immunopharmacol..

[157] Geng X., Wu H., Li Z., Li C., Chen D., Zong J., Liu Z., Wei S., Peng W. (2021). Jie-Yu-He-Huan Capsule Ameliorates Anxiety-Like Behaviours in Rats Exposed to Chronic Restraint Stress via the cAMP/PKA/CREB/BDNF Signalling Pathway. Oxidative Med. Cell. Longev..

[158] Akter S., Sasaki H., Uddin K.R., Ikeda Y., Miyakawa H., Shibata S. (2019). Anxiolytic effects of γ-oryzanol in chronically- stressed mice are related to monoamine levels in the brain. Life Sci..

[159] Satao K., Borkar M., Godad A., Doshi G. (2026). Repurposing of bithionol as a potential anxiolytic agent through NF-κB suppression: Insights from behavioural, biochemical, and molecular modelling studies. Toxicol. Appl. Pharmacol..

[160] Di Liberto V., Frinchi M., Verdi V., Vitale A., Plescia F., Cannizzaro C., Massenti M.F., Belluardo N., Mudò G. (2017). Anxiolytic effects of muscarinic acetylcholine receptors agonist oxotremorine in chronically stressed rats and related changes in BDNF and FGF2 levels in the hippocampus and prefrontal cortex. Psychopharmacology.

[161] de Siqueira Patriota L.L., de Lima B.R.F., de Oliveira Marinho A., da Costa J.A., de Lucena A.L.A., Paiva P.M.G., Napoleão D.C., Cavalcanti J.V.F.L., Pereira M.C., Napoleão T.H. (2025). Moringa oleifera Lam. seed lectin (WSMoL) reduces chronic stress-induced anxiety and depression in mice by lessening inflammation and balancing brain chemicals. Behav. Brain Res..

[162] Zhou J., Wang J., Li W., Wang C., Wu L., Zhang J. (2017). Paeoniflorin attenuates the neuroinflammatory response in a rat model of chronic constriction injury. Mol. Med. Rep..

[163] Bai H., Chen S., Yuan T., Xu D., Cui S., Li X. (2021). Paeoniflorin ameliorates neuropathic pain-induced depression-like behaviors in mice by inhibiting hippocampal neuroinflammation activated via TLR4/NF-κB pathway. Korean J. Physiol. Pharmacol..

[164] Cheng C., Lin J.Z., Li L., Yang J.L., Jia W.W., Huang Y.H., Du F.-F., Wang F.-Q., Li M.-J., Li Y.-F. (2016). Pharmacokinetics and disposition of monoterpene glycosides derived from Paeonia lactiflora roots (Chishao) after intravenous dosing of antiseptic XueBiJing injection in human subjects and rats. Acta Pharmacol. Sin..

[165] Nutma E., Fancy N., Weinert M., Tsartsalis S., Marzin M.C., Muirhead R.C.J., Falk I., Breur M., de Bruin J., Hollaus D. (2023). Translocator protein is a marker of activated microglia in rodent models but not human neurodegenerative diseases. Nat. Commun..

[166] Kéri S., Kancsev A., Kelemen O. (2024). Algorithm-Based Modular Psychotherapy Alleviates Brain Inflammation in Generalized Anxiety Disorder. Life.

[167] Kurtys E., Doorduin J., Eisel U.L., Dierckx R.A., de Vries E.F. (2017). Evaluating [11C]PBR28 PET for Monitoring Gut and Brain Inflammation in a Rat Model of Chemically Induced Colitis. Mol. Imaging Biol..

[168] Cho E.J., Kim H.Y., Lee A.Y. (2020). Paeoniflorin ameliorates Aβ-stimulated neuroinflammation via regulation of NF-κB signaling pathway and Aβ degradation in C6 glial cells. Nutr. Res. Pract..

[169] Guo X., Han Z., Sun J., Liu S., Zhang C., Xu G., Wang X., Song Q., Yang H., Li A. (2025). Paeoniflorin Alleviates LPS-Induced Inflammation and Acute Myocardial Injury by Inhibiting PI3K/Akt/ERK-Mediated HIF-1α and TLR4/MyD88/NF-κB Inflammatory Signaling. Mediat. Inflamm..

[170] Nie X.H., Ou-yang J., Xing Y., Li D.Y., Dong X.Y., Liu R.E., Xu R. (2015). Paeoniflorin inhibits human glioma cells via STAT3 degradation by the ubiquitin-proteasome pathway. Drug Des. Dev. Ther..

[171] Zhang Z., Yang W. (2021). Paeoniflorin protects PC12 cells from oxygen-glucose deprivation/reoxygenation-induced injury via activating JAK2/STAT3 signaling. Exp. Ther. Med..

[172] Wang X., Jiang L., Liu X.Q., Huang Y.B., Wang A.L., Zeng H.X., Gao L., Zhu Q.-J., Xia L.-L., Wu Y.-G. (2022). Paeoniflorin binds to VEGFR2 to restore autophagy and inhibit apoptosis for podocyte protection in diabetic kidney disease through PI3K-AKT signaling pathway. Phytomedicine.

[173] Guo R.B., Wang G.F., Zhao A.P., Gu J., Sun X.L., Hu G. (2012). Paeoniflorin protects against ischemia-induced brain damages in rats via inhibiting MAPKs/NF-κB-mediated inflammatory responses. PLoS ONE.

[174] Xie W., Yu W., Zhao M., Zhou W., Chen H., Du W., Huang L., Xu Y., Li X. (2011). Protective effect of paeoniflorin against oxidative stress in human retinal pigment epithelium in vitro. Mol. Vis..

[175] Cong C., Kluwe L., Li S., Liu X., Liu Y., Liu H., Gui W., Liu T., Xu L. (2019). Paeoniflorin inhibits tributyltin chloride-induced apoptosis in hypothalamic neurons via inhibition of MKK4-JNK signaling pathway. J. Ethnopharmacol..

[176] Huang C., Zhang X., Yu J., Xuan C., Xu C., Li X., Cui H., Xu D. (2026). Paeoniflorin mitigates hyperoxia-induced cognitive deficits in neonatal mice by modulating p38/JNK signaling to reduce oxidative stress and neuroinflammation. Neuropharmacology.

[177] Zhou J., Wang L., Wang J., Wang C., Yang Z., Wang C., Zhu Y., Zhang J. (2016). Paeoniflorin and Albiflorin Attenuate Neuropathic Pain via MAPK Pathway in Chronic Constriction Injury Rats. Evid.-Based Complement. Altern. Med..

[178] Liu Y., Han J., Zhou Z., Li D. (2019). Paeoniflorin protects pancreatic β cells from STZ-induced damage through inhibition of the p38 MAPK and JNK signaling pathways. Eur. J. Pharmacol..

[179] Li W., Zhi W., Liu F., Zhao J., Yao Q., Niu X. (2018). Paeoniflorin inhibits VSMCs proliferation and migration by arresting cell cycle and activating HO-1 through MAPKs and NF-κB pathway. Int. Immunopharmacol..

[180] Guo W., Yao X., Cui R., Yang W., Wang L. (2022). Mechanisms of paeoniaceae action as an antidepressant. Front. Pharmacol..

[181] Hou Y., Li H., Zhu L., Quan T., Feng X., Li Y., Bian Y., Wei Y. (2025). Advances of paeoniflorin in depression: The molecular mechanism and formula application. Front. Pharmacol..

